# Structural Plasticity on the SpiNNaker Many-Core Neuromorphic System

**DOI:** 10.3389/fnins.2018.00434

**Published:** 2018-07-02

**Authors:** Petruț A. Bogdan, Andrew G. D. Rowley, Oliver Rhodes, Steve B. Furber

**Affiliations:** School of Computer Science, University of Manchester, Manchester, United Kingdom

**Keywords:** SpiNNaker, structural synaptic plasticity, synaptic rewiring, neuromorphic computing, spiking neural networks, topographic map, synaptogenesis

## Abstract

The structural organization of cortical areas is not random, with topographic maps commonplace in sensory processing centers. This topographical organization allows optimal wiring between neurons, multimodal sensory integration, and performs input dimensionality reduction. In this work, a model of topographic map formation is implemented on the SpiNNaker neuromorphic platform, running in realtime using point neurons, and making use of both synaptic rewiring and spike-timing dependent plasticity (STDP). In agreement with Bamford et al. (2010), we demonstrate that synaptic rewiring refines an initially rough topographic map over and beyond the ability of STDP, and that input selectivity learnt through STDP is embedded into the network connectivity through rewiring. Moreover, we show the presented model can be used to generate topographic maps between layers of neurons with minimal initial connectivity, and stabilize mappings which would otherwise be unstable through the inclusion of lateral inhibition.

## 1. Introduction

Ramón y Cajal postulated that: “In the adult centers, the nerve paths are something fixed, ended, and immutable. Everything may die, nothing may be regenerated” (Ramón y Cajal, 1928). We now know that not to be the case. Mammalian brains change their connectivity from early development and throughout adulthood. Perinatally, the neuromuscular junction sees neural competition for the innervation of muscle fibres resulting in their receiving inputs from single motoneurons (Buffelli et al., 2004; Favero et al., 2010). Postnatally, brains undergo a period of over-growth of synapses which is maintained until puberty when massive synaptic pruning occurs (Zecevic and Rakic, 1991).

Connectivity changes to brains are not only limited to development, they occur throughout an adult's life. Synaptic rewiring occurs in ischemic areas to recover function (Butz and van Ooyen, 2013; Mascaro et al., 2016), in the reward center of the brain as a result of drug addiction (Robinson and Kolb, 1997; Russo et al., 2010), in neurogenic areas in order to functionally integrate newborn neurons (Lledo et al., 2006), during learning (Benson et al., 2001; Holtmaat et al., 2005; Xu et al., 2009), memory formation and long term storage, in which it plays a central role (Poirazi and Mel, 2001; Kleim et al., 2002; Lamprecht and LeDoux, 2004), and during enriched experiences (Van Ooyen and Butz-Ostendorf, 2017). There is tight interplay between structural changes in the connectivity between neurons and the efficacies of existing connections. When viewed at the microscopic level, the projections of cortical neurons are so crowded that they can essentially be viewed as a potential all-to-all connectivity; a potential connection is one in which the growth of a spine or terminal bouton could form a synapse (Hellwig, 2000; Kalisman et al., 2005). However, not all potential connections are formed; the local microcircuitry of the cortex is functionally highly selective and generally maintains a sparse connectivity (Stepanyants et al., 2002; Le Bé et al., 2006). Synaptic plasticity mechanisms such as spike-timing dependent plasticity, which cause long-term potentiation or depression (Markram et al., 1997; Bi and Poo, 1998), have been reported to be closely linked to structural changes (Le Bé et al., 2006; Holtmaat and Svoboda, 2009).

In terms of structural plasticity, the research focus of computational neuroscience typically lies on synaptic rewiring, thus this paper will not discuss the creation of new neurons as a form of structural plasticity. In the remainder of the paper, “structural plasticity” is used in lieu of structural synaptic plasticity, and can be used interchangeably with “synaptic rewiring.”

### 1.1. Contributions

In this paper we implement the model proposed by Bamford et al. (2010) (described in detail in section 2.2.2) using a novel structural plasticity framework designed for the SpiNNaker system (section 2.1). We make use of the speed-up achieved by running the model in real time on SpiNNaker to explore whether it is suitable for modeling developmental formation of topographic maps over longer time-scales (section 3.3). We explore the behavior of the network when excitatory lateral connections are replaced with inhibitory ones, revealing the stabilizing effect these have on the network (exploration of this effect beginning in section 3.4). Finally, we perform sensitivity analysis on the network to establish an operational range of various parameters. We show that: (1) SpiNNaker is well-suited for parameter sweeps and even hyperparameter optimization due to its massive parallelism, and (2) the network has low inter-trial variability, except in certain scenarios discussed in section 3.5.

SpiNNaker is a general-purpose neuromorphic platform with a sizeable user base. The model described here has been implemented so as to allow use as an “off-the-shelf” learning mechanism. The Python scripts and data generated from executing the simulations on SpiNNaker are available online^1^.

### 1.2. Computational models of structural plasticity

Structural synaptic plasticity is an omnipresent mechanism in mammalian brains, involved in learning, memory, and recovery from lesions. Structural plasticity in the form of synaptic rewiring is also a useful computational tool, used to automatically generate connectivity based on experimental activity data (Diaz-Pier et al., 2016), explore network states for Bayesian inference (Kappel et al., 2015, 2018), assist synaptic plasticity rules to achieve better performance (Spiess et al., 2016), allocate limited computational resources optimally (George et al., 2017), and more efficiently and accurately encode patterns (Poirazi and Mel, 2001; Roy et al., 2014; Hawkins and Ahmad, 2016; Roy and Basu, 2017), to name a few.

Diaz-Pier et al. (2016) view structural plasticity as a mechanism for network optimization. In a time when neural activity data is abundant, but connectivity data is sparse, and when network models are mostly hand crafted, they use the structural plasticity model proposed by Butz and van Ooyen (2013) to achieve a desired mean activity level for the network through automatic self-organization. The local homeostatic mechanism rewires neurons in a network based on their mean spiking activity, their available dendritic and axonal connection points, and the distance between them. This structural plasticity mechanism is able to account for cortical reorganization after deafferentiation and stroke. Both of these models were run on the NEST simulator (Bos et al.,^2015^).

Structural plasticity models need not include both formation and deletion rules. Iglesias et al. (2005) simulates the effect of massive synaptic pruning resulting after an initial developmental overproduction of synapses, similar to those explored *in vivo* by Zecevic and Rakic (1991). They show that, even without continuous formation of new synapses, a network will stabilize at approximately 10% of the starting number of active synapses, regardless of network size. This relies heavily on the choice of deletion rule, which must be very conservative, i.e., only prune synapses with no chance of becoming active.

Kappel et al. (2015) propose a mathematical view of structural plasticity from the perspective of Bayesian inference. Their networks sample parameters from a prior distribution to obtain desirable characteristics, such as sparse connectivity and heavy-tailed distributions of synaptic weights. Moreover, they explain that stochastic dynamics of network parameters, which cause trial-to-trial variability in experiments, should be viewed as a “functionally important component of the organization of network learning.” They subsequently they show that the underlying stochasticity of neuronal networks allows the exploration of various network configurations while maintaining its functionality. In a follow-up paper, Kappel et al. (2018) extend their framework with the introduction of reward driven reorganization, operating side-by-side with synaptic plasticity. They replicate experimental results which observe task-dependent reorganization of connections between the cortex and basal ganglia. Importantly, their model takes into account recent findings that a significant amount of synaptic and structural plasticity happens stochastically, not solely based on activity (Dvorkin and Ziv, 2016).

Structural plasticity models need not include both formation and deletion rules. Iglesias et al. (2005) simulates the effect of massive synaptic pruning resulting after an initial developmental overproduction of synapses, similar to those explored in vivo by Zecevic and Rakic (1991). They show that, even without continuous formation of new synapses, a network will stabilize at approximately 10% of the starting number of active synapses, regardless of network size. This relies heavily on the choice of deletion rule, which must be very conservative, i.e., only prune synapses with no chance of becoming active.

Structural plasticity has also been shown to aid in denoising the response of a network, and improve the learning speed of synaptic plasticity mechanisms. Spiess et al. (2016) designed a model of structural plasticity which strives to maintain a constant number of synapses. Overlaid on top is a “pruning” mechanism, which steadily decreases the target number of synapses. Within their regime, they see a halving of the required time for the network to learn the presented patterns.

Modeling work by Poirazi and Mel (2001) showed that neurons with nonlinear dendrites have larger information storage capacity compared to those with linear dendrites. Moreover, they believe that long-term information storage rests in the connectivity to the dendritic sub-units, rather than in the weights of connections. This work has been extended in subsequent years by Roy et al. (2014) in the context of liquid state machines (LSM, Maass and Markram, 2004) to achieve efficient readout. Here, the gradient descent-driven synaptic rewiring rule is applied to the readout network to minimize error. They computed a weight modification for each cell, but rather than applying it to the weights it is used to drive rewiring: a low value of the fitness function results in the synapse being marked for replacement with a more performant one. The versatility of the two-compartment unit with the addition of a suitable structural plasticity rule was also proven within winner takes all (WTA) networks (Roy and Basu, 2017). Both of the previous experiments are characterized by the application of synaptic rewiring at the end of pattern presentations.

The system of Hawkins and Ahmad (2016) (Hierarchical Temporal Memory) uses sparse encoding of sensory inputs to learn a sequence of patterns (Olshausen and Field, 2004). Neurons are attached to some number of coincidence detectors, relying on the existence of a set of co-located synapses that connect to a subset of the cells that are active in the pattern to be recognized. When attempting to learn a new pattern, new synapses are formed. These synapses have a permanence value which represents their growth; the weight of the synapse is binary and derived from the permanence. A high permanence means the synapse is active, otherwise the synapse is reassigned.

Recent work by George et al. (2017) implementing synaptic rewiring in neuromorphic hardware showed that structural plasticity enables optimal computational resource allocation. Their rule requires the use of an FPGA co-processor to the Reconfigurable On-Line Learning Spiking device (ROLLS, Qiao et al., 2015); the co-processor drives the rewiring of synapses between point neurons by periodically inspecting the weights of connections against a threshold. In their model, synapses are pruned if they have been depressed, i.e., their weights are bellow a threshold, for a fixed amount of time; after pruning, a new connection is immediately formed.

Bamford et al. (2010) used structural plasticity to reduce receptive fields of point neurons when modeling the formation of neuronal topographic maps. Their model also uses STDP alongside the formation and elimination of synapses. Their model performed a fixed number of rewiring attempts in each simulation time step with exactly one of three actions being performed: formation of a new synapse, removal of an older synapse or no action; synapses are not replaced instantaneously and all actions are probabilistic. They saw a reduction in the spatial variance of neuronal receptive fields, while also preserving the desired position of the centers of these receptive fields. Finally, the input selectivity learnt through STDP was embedded into the network connectivity by the structural plasticity mechanism.

### 1.3. SpiNNaker neuromorphic computing platform

Furber et al. (2013, 2014) designed and built the SpiNNaker neuromorphic computing platform. It is a many-core machine making use of 18 ARM968 processors per chip, operating at around 200 MHz and with 64 MBytes of local data memory, 32 MBytes of local instruction memory and 128 MBytes of SDRAM shared across all cores on the chip. Each chip connects to 6 of its neighboring chips via the SpiNNaker router. The SpiNNaker network communicates between the chips and cores using small packets which contain either 32 or 64 bits of data following the Address Event Representation (AER, Deiss et al., 1999). SpiNNaker chips are organized into boards of 48-chips which are then joined together, allowing up to 1 million processors to run and communicate in parallel in a single simulation, making it one of the largest neuromorphic platforms currently in existence (Furber, 2016).

The SpiNNaker platform was designed to simulate networks of simple spiking neurons, with each core theoretically capable of simulating up to 1,000 neurons, each with 1,000 synaptic inputs. When simulated with a time step of 1 ms between updates of the neural state, the network can then run in real time. Thus, when a large number of cores are running simultaneously, large networks can be simulated that would otherwise run much slower on conventional computing systems. This makes the system ideal for neurorobotic applications where the systems need to respond to stimuli in real time.

The SpiNNaker communication network allows spike packets to be transmitted to multiple cores simulating neurons near-simultaneously across the entire machine, guaranteeing that the messages arrive within less than the 1 ms simulation time step (Navaridas et al., 2009). This is made possible through the support of multicast within the router on each chip, which allows it to forward a received packet to any or all of the neighboring chips and local cores in a single clock-tick.

The real-time constraint is one of two design drivers of SpiNNaker, the other being energy efficiency. SpiNNaker uses the RISC architecture of ARM processors with local, attached memory, coupled with protocols which turn off application cores when not in use. SpiNNaker makes use of an interrupt-driven computational system, which is very desirable when optimizing for energy efficiency. Additionally, SpiNNaker focuses on simulating “point neuron models,” morphological simplifications of a neuron wherein the details of their dendritic structure is ignored; synapses between neurons are simulated as being formed directly onto the soma.

### 1.4. Simulation software on spiNNaker

SpiNNaker uses an event-driven computation model when computing synaptic and neuron updates (Brown et al., 2015). An incoming spike (packet event) is placed in a buffer, which triggers a direct memory access (DMA) read of the SDRAM memory region which contains synaptic information (weights, delays etc.). The synaptic processing is performed along with any spike-timing-dependent-plasticity updates, and the data is then written back the the SDRAM through another DMA. The neuron update is performed on a timer interrupt which occurs every simulation time step.

PyNN is a simulator-independent language for describing spiking neural network models created by Davison et al. (2008). PyNN is used as a front-end to the SpiNNaker system, which make it easier for users to describe their networks without having to interact directly with the SpiNNaker hardware. The SpiNNaker-specific PyNN implementation (sPyNNaker, Stokes et al., 2016) controls how neuronal populations are partitioned and placed onto individual SpiNNaker cores and sets up the on-chip routers so as to allow multicast communication between chips (Mundy et al., 2016). The software then converts the neural network parameters and connectivity data into a form that the cores can make use of when performing their updates.

### 1.5. Current status of learning on spiNNaker

Learning on SpiNNaker is currently implemented in the form of long-term potentiation/depression (LTP/LTD) instigated via spike-timing-dependent plasticity (STDP). From the perspective of a synapse, the relative timing of pre- and post-synaptic action potentials is used as a measure of causality and forms the basis of a synaptic weight change. Hebbian two-factor learning is employed (Song et al., 2000), where a pre-synaptic spike, shortly followed by a post-synaptic spike is identified as a causal relationship, and potentiated; conversely, a post-pre pairing is depressed (Markram et al., 1997; Bi and Poo, 1998).

A range of STDP learning rules is implemented within the sPyNNaker API, with the implementation framework following the modular structure of PyNN. Individual timing and weight update rules are specified on a per projection basis, where a projection is defined as a directional link between populations of neurons. Timing rules operate on pre- and post-synaptic history traces, and include the classical spike pair rule, the triplet rule described by Pfister and Gerstner (2006), and the homeostatic rule designed by Vogels et al. (2011). Weight updates based on the results from timing assessment can be made either in additive or multiplicative form, following the methods summarized in Morrison et al. (2008).

The implementation has been designed with chip architecture and performance in mind, with the goal of real-time simulation of neural networks containing plastic synapses. As the synapse input is processed on the post-synaptic core, a challenge presented by the hardware is the restricted information available with respect to the synapse structure and connectivity (Diehl and Cook, 2014). Whilst a neuron processing core stores neuron state variables in local memory, the comprehensive synaptic connectivity data is relatively large, and hence must be stored in shared memory. When a neuron receives a spike, the appropriate synaptic data is transferred from shared to local memory, enabling the appropriate weight contribution to be made to the neuron input. For efficiency and convenience, plastic weight updates are therefore limited to pre-synaptic events (i.e., receiving a spike), as it is at these times the synaptic weight has been transferred into local memory for processing. Pre-synaptic traces are stored alongside synaptic data in shared memory (and retrieved and updated on spike arrival at the post-synaptic neuron); whilst post-synaptic trace histories are maintained between pre-synaptic events in core-local memory. This framework, known as the deferred event driven (DED) model (Jin et al., 2010), provides all the information necessary to perform synaptic updates based on relative spike timing.

## 2. Materials and methods

The current section is concerned with providing an overview of the SpiNNaker structural plasticity framework (section 2.1) that was created in order to facilitate the implementation of the model of synaptic rewiring proposed by Bamford et al. (2010) (section 2.2). Finally, the analysis methodology is defined in section 2.3.

### 2.1. Structural plasticity framework

SpiNNaker's software has been designed with object oriented programming in mind even though the language used at the lowest level is C. The structural plasticity framework functions in a similar fashion to the current plasticity rules, but with different dependencies. After the initialization, a batch of rewiring attempts is triggered every time step which can result in either a new synapse being formed, a synapse being eliminated or the attempt being aborted; the formation and deletion functions are called as a result of DMA callbacks.

The structural plasticity framework required additional local information compared to the present STDP learning rules. STDP has access to connectivity information, weights, delays and synapse types, which are all contained in a sparse matrix. This synaptic matrix consists of rows indexed by pre-synaptic neuron. The default information present in the synaptic matrix is not sufficient to identify pre-synaptic neurons in space, or even whether synaptic rewiring is enabled for that specific set of connections. Additional metadata must be loaded into core-local memory. Each core responsible for performing synaptic rewiring is provided with additional information about the population as a whole:the shape of the grid and the total number of neurons, but also some information about itself as a part of the population. This information is sufficient for a neuron's position to be established. Currently, the same grid shape is used for all populations connected to the structurally plastic population. Scalability is impacted with the introduction of metadata items which represent individual pre-synaptic entities. These issues and their possible solutions are discussed in section 4.1.

The present STDP learning rules have been adapted to interface seamlessly with the structural plasticity mechanism. Operations on synaptic rows have to be performed by the individual learning rules, as the rows may contain rule-specific information, data formats or headers. Therefore, for the rules to support synaptic rewiring, they have been augmented with three functions: searching for a post-synaptic neuron in the row, adding, and removing a post-synaptic neuron to the row. The decision of which synapses to form or remove has to be defined within the model implemented using the framework presented here.

To summarize, the requirements for a synaptic plasticity mechanism to function alongside structural plasticity is to implement the three previously described functions: find, add, and remove post-synaptic neurons. Conversely, a novel model of structural plasticity need not define all presented methods. For example, a model solely responsible for pruning, akin to that designed by Iglesias et al. (2005), need only implement a rule for removing synapses. However, models relying on additional information, for example on the firing rate of neurons or the extra-cellular concentration of some neurotransmitter would require more profound changes be performed. For example, assuming the use of a leaky integrate-and-fire (LIF) neuron, the homeostatic structural plasticity model proposed by Butz and van Ooyen (2013) would necessitate the modification of the LIF neuron to report its time averaged firing rate. If considering a neuromodulated STDP rule (Mikaitis et al., 2018), the operation of the rewiring rules might be as presented in the following sections, but it would be conceivable that the reporting of instantaneous dopamine concentration could transiently alter rewiring parameters (frequency of rewiring, probabilities involved in the computation).

### 2.2. Computational model of topographic map formation

#### 2.2.1. Overview

Topographic maps are areas of the brain where neural responses vary continuously across the area. Additionally, a topographic mapping is formed by neurons from one area projecting to those in another area such that neighboring neurons in the target area are maximally responsive to the activity of a neighborhood in the source area. Topographic maps seem to be the representation of choice throughout mammalian brains, being present in the somatosensory cortex as well as the visual cortex (Kaas, 1997) and even olfactory bulb (Mombaerts et al., 1996). Despite their ubiquity, their purposes is still debated, with potential benefits ranging from wiring efficiency, to dimensionality reduction (Chklovskii and Koulakov, 2004) and multimodal integration (Holmes and Spence, 2005).

The computational model designed and implemented by Bamford et al. (2010) aimed to explore some properties of topographic maps and their formation. The model was deliberately designed to be as generic as possible, while also fitting within the constraints of implementing in VLSI. Their model has been chosen for the basis of the presented framework and implementation because: (1) it makes use of synaptic learning rules and neuron models which are readily available on SpiNNaker, (2) it proposes a good basis for expansion and application in a model of neurogenesis, and (3) it is a model with particularly desirable qualitative features:

- Refines an initially rough topographic map
- Embeds input preferences into the connectivity of the network
- Simple enough to run in real time, alongside other learning rules
- Powerful enough that the network develops appropriate ocular preference when presented with binocular input.

**Figure 1:**
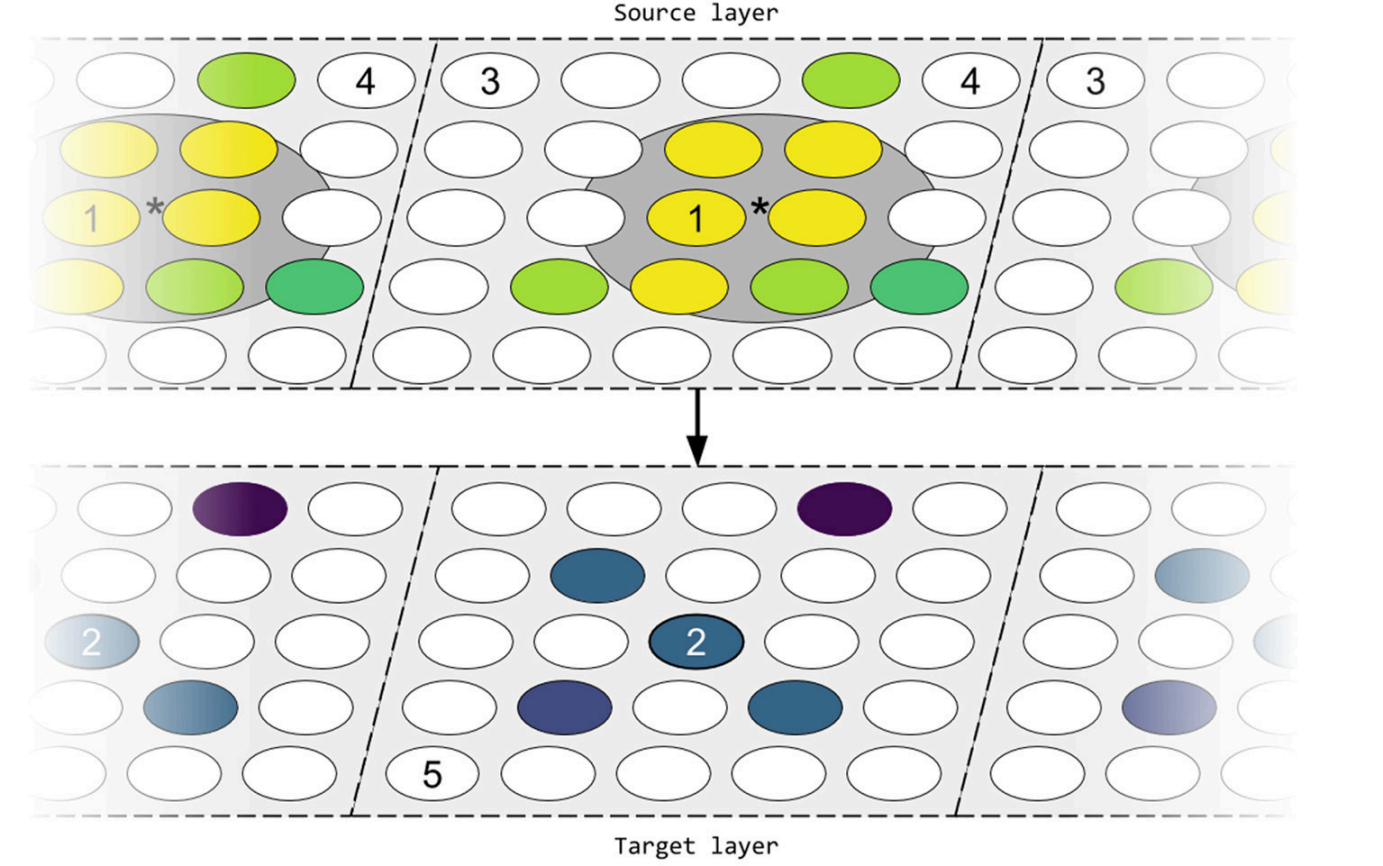
Simplified network architecture. Neurons are placed on a rectangular mesh at discrete locations. Neuron 2 in the target layer has a receptive field formed by the connections from the source layer (feedforward), as well as connections coming from within the target layer (lateral). These connections will ideally be centred around the spatially closest neuron (the ideal location), i.e., neuron 1 in the case of feedforward connections, or around itself for lateral connections. In practice, a limited number of synapses means that the center of the receptive field (^*^, computed as the location which minimizes the weighted variance of the feedforward connections) will deviate from the ideal location. Additionally, connections from more distant neurons are likely to decrease in strength (darker color equates with weaker connections); cells in white do not connect to neuron 2. The topology of the network is toroidal; periodic boundary conditions are denoted by dashed edges and the layer tessellation. Here, the distance between neurons 3 and 4 in source layer is equal to the distance between neurons 3 and 5 in the source and target layer respectively (1 unit).

The simulated model focuses on the refinement process affecting the connectivity between two layers of neurons with periodic boundary conditions (PBC), as seen in Figure 1. Topographic map refinement is reported via two metrics: the spatial spread of the mean receptive field (σ_*aff*_); and the mean deviation of the center of the receptive fields from the ideal location (*AD*). The measure of distance is thus crucial for defining these metrics. The Euclidean distance δ between neurons is computed in relation to the PBC and assuming no spatial separation between layers. We define the ideal location for the receptive field of a neuron as the location at distance δ = 0 from the neuron; in Figure 1 neuron 2 has a feedforward receptive field which would ideally be centred on neuron 1, and a lateral receptive field which would ideally be centred around itself.

After simulating the network for some period of time we observe that, in general, the mean receptive field of target-layer neurons originates from a more spatially clustered group of source-layer neurons and that the centers of these fields will, on average, only slightly deviate from their ideal positions. The non-zero deviation occurs as a consequence of the relatively few synapses which sample the area around the ideal location. These metrics are considered in three types of experiments:

- Case 1: STDP and rewiring operating simultaneously in the presence of correlated input
- Case 2: STDP but no rewiring in the presence of correlated input
- Case 3: STDP and rewiring without correlated input

These experiments have been chosen to reveal the role of synaptic rewiring and input correlations on the quality of topographic maps. Herein we also explore whether the mechanism is capable of developing a topographic map, the effect of lateral inhibition and parameter sweeps in terms of the same three cases. The creation and refinement of these topographic mappings is shown to be a result of the co-operation of two continuously operating and parallel plasticity mechanisms: STDP and synaptic rewiring.

The proposed variant of synaptic rewiring consists of two rules:

1. A distance-dependent rule for creating connections (activity-independent formation rule)
2. A weight-dependent rule for removing connections (activity-dependent removal rule)

Intuitively, single neurons from the target layer will, on average, form more connections with the closest neurons to them. These connections will, by themselves, fully define a rough topographic mapping. Whether this is taken to be the initial connectivity of the network makes little difference to the quality of the final, refined, mapping as long as this process also occurs continuously and in concert with an activity-dependent mechanism. Under the assumption that connections with lower weights are weaker, thus more unstable and prone to pruning, a spike-timing dependent plasticity rule was chosen which potentiates causal low-latency connections, i.e., feedforward connections, while higher-latency lateral connections are depressed. The presence of an elimination rule which preferentially targets depressed synapses results in a tightening of the neuronal receptive field which is subsequently embedded into the connectivity the network.

The model presented here differs from recent work in several salient respects. Synapses with continuous weights are fundamental for the correct operation of the model as presented. Alterations allowing for association between synapses and values encoding permanence (Hawkins and Ahmad, 2016), fitness (Roy and Basu, 2017) or virtual weights (George et al., 2017) are possible, but these are not explored herein. Moreover, the proposed rewiring rules operate on point neurons. In this respect, it is similar to the model proposed by George et al. (2017), though it differs in the choice of differential equations which represent the neurons.

Additionally, our proposed rule differs in the choice of pre-synaptic partner selection mechanism. Herein we propose that the synapse formation rule selects one of the last neurons to have spiked in the past time step as we lack the time resolution to distinguish between spikes generated in the same time step. This is a departure from the selection protocol of Bamford et al. (2010) who selected the last neuron to have spiked; their approach was less affected by this choice as their time step was 0.1 ms and the probability of multiple co-occurring spikes was lower. Other approaches involve forming new connections randomly around the strongest connection (George et al., 2017), around the neuron (Butz and van Ooyen, 2013) or by considering a set of potential performant synapses (Roy et al., 2014; Hawkins and Ahmad, 2016; Roy and Basu, 2017). Finally, rewiring attempts occur continuously throughout the simulation (Kappel et al., 2015; Hawkins and Ahmad, 2016; George et al., 2017), as opposed to happening in a batch at the end of a pattern presentation (Roy et al., 2014; Roy and Basu, 2017) or at regular discrete intervals (Butz and van Ooyen, 2013).

The remainder of section 2.2 includes: a detailed description of the synaptic rewiring mechanism, neuron and synapse models, experimental parameters, initial conditions and input generation, and a presentation of the analysis methods.

#### 2.2.2. Details of the model

The model is currently defined in relation to two layers of neurons: a source and target. Connections are formed from neurons in the source to those in the target layer, as well as from neurons in the target layer to others in the same layer. Neurons are placed at discrete locations on a two-dimensional mesh for efficient distance computation with periodic boundary conditions to avoid artefacts affecting neurons positioned on the edge of the layer.

The goal of the model is to create a topographic mapping between the two layers using two continuously operating plasticity mechanisms: synaptic plasticity or adjusting the weights of existing connections between neurons, and structural plasticity or adjusting the connectivity between neurons. No assumptions about the initial connectivity need to be made. A further sub-division of the mechanisms allows categories of processes to be identified: activity-independent and activity-dependent processes. An activity-independent process guides axonal growth to the “ideal” location in the target area. Axonal branching results in the formation of synapses in the area surrounding the ideal topographic location.

Further, a competitive Hebbian process detects input correlations due to the spatial proximity of synapses in the source layer. Thus, synapses arriving from spatially clustered neurons will be strengthened, while synapses from neurons which are more spatially separated will be weakened. As a result of this activity-dependent processes, the effective spread of the target-layer neural receptive fields is reduced. This effect can then be amplified by the continuous preferential removal of weak synapses. In this fashion, the reduction of the receptive fields is embedded into the network topology. However, when formation and removal are continuously applied there is potential for the receptive fields to be refined further.

The model used in the rest of the paper is defined between two layers of *N*_*layer*_ neurons equidistantly placed on a square mesh with PBC (as seen in Figure 1), though the implementation allows definition of any rectangular grid. Initially, the two layers are connected either using a fully-defined topographic map for the purpose of refinement (Bamford et al., 2010), an arbitrary percentage-based connectivity (George et al., 2017), or no connectivity.

Each target layer neuron has a fixed maximum fan-in or synaptic capacity *S*_*max*_ – feedforward connections compete with recurrent, lateral connections for these synaptic slots. If a neuron does not receive the maximum allowed number of afferent connections it is said to have some number of potential synapses. With a fixed frequency *f*_*rew*_, a random synaptic element is chosen from a target-layer neuron. If the selected element is a potential synapse, i.e., no connection currently exists, the formation rule is followed: a partner pre-synaptic cell is selected and the Euclidean distance between the two cells is computed. Finally, a new, full-strength, connection will be formed if

(1) $$\begin{array}{l} r<p_{\text{form}}e^{-\frac{\delta^{2}}{2\sigma_{\text{form}}^{2}}} \end{array}$$

where *r* is a random number sampled from a uniform distribution in the interval [0, 1), *p*_*form*_ is the peak formation probability, δ is the distance between the two cells and $\sigma_{\mathrm{form}}^{2}$ is the variance of the receptive field. The result is a Gaussian distribution of formed synapses around the ideal target site, i.e., around the target neuron where δ = 0. The same formation rule is followed by lateral connections and defines an initial rough topographic mapping.

If the randomly selected synaptic element exists, the removal rule is followed. For implementation efficiency and because of the nature of the synaptic plasticity rule (weight-independent STDP), a weight threshold θ_*g*_ is selected as half of the maximum allowed weight ($\theta_{g}=\frac{1}{2}g_{\max}$).

(2) $$\begin{array}{l} r<p_{\text{elim}}\;\text{where}\;p_{\text{elim}}=\{\begin{array}{cc} p_{\text{elim}-\text{dep}}\; & \mathrm{for}\;g_{\text{syn}}<\theta_{g}\\ p_{\text{elim}-\text{pot}}\; & \mathrm{for}\;g_{\text{syn}}\geq \theta_{g}\\ \; \end{array} \end{array}$$

where *r* is a random number sampled from a uniform distribution in the interval [0, 1), *p*_*elim*−*dep*_ is the elimination probability used when a synapse is depressed, *p*_*elim*−*pot*_ is the elimination probability used when a synapse is potentiated and *g*_*syn*_ is the weight of the synapse under consideration for removal.

The SpiNNaker implementation is detailed in Algorithm 1 is adapted from Bamford et al. (2010) and describes the model as it runs on SpiNNaker. The parameters provided in Table 1 are written into shared memory (SDRAM). The formation rule (Equation 1) is translated into an integer lookup table (LUT) for each type of connection (feedforward and lateral) and also written to SDRAM. This is because the computation includes an computationally expensive exponential decay term and SpiNNaker does not have a floating point unit. At initialisation, the parameters and probability LUTs are copied into core-local memory (DTCM), while pointers are created to locate the neuronal synaptic elements in SDRAM. The later table contains information relating a post-synaptic neuron to its pre-synaptic partners; this is complementary to the synaptic matrix which is designed to relate an AER spike to post-synaptic partners. Finally, we ensure the prescribed frequency of rewiring *f*_*rew*_ by using a shared seed for synchronisation.

**Algoritham 1:**
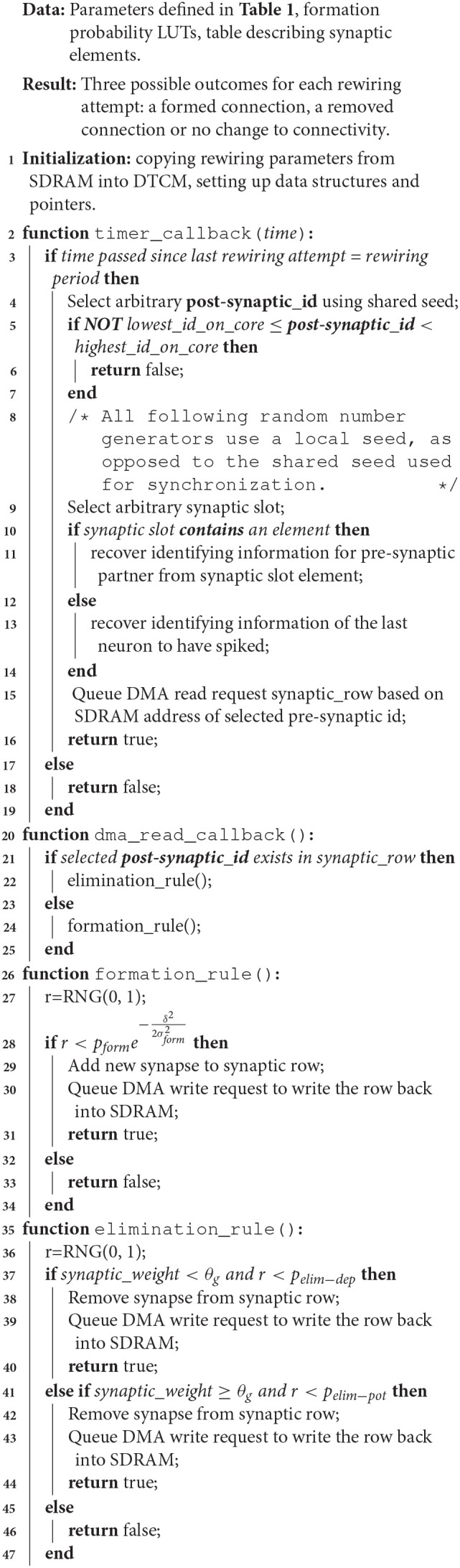
Algorithmic description of the synaptic rewiring model running on SpiNNaker.

**Table 1:** Simulation parameters.

**Wiring**	**Inputs**	**Membrane**	**STDP**	**Simulation**
*N*_*layer*_ = 16 × 16	*f*_*mean*_ = 20 Hz	*V*_*rest*_ = −70 mV	*A*_+_ = 0.1	Δ*t* = 1 ms
*S*_*max*_ = 32	*f*_*base*_ = 5 Hz	*E*_*ext*_ = 0 mV	*B* = 1.2	timescale = 1
σ_*form*−*ff*_ = 2.5	*f*_*peak*_ = 152.8 Hz	*V*_*thr*_ = −54 mV	τ_+_ = 20 ms	
σ_*form*−*lat*_ = 1	σ_*stim*_ = 2	*g*_*max*_ = 0.2	τ_−_ = 64 ms	
*p*_*form*−*ff*_ = 0.16	*t*_*stim*_ = 20 ms	τ_*m*_ = 20 ms	*w*_*min*_ = 0 mS	
*p*_*form*−*lat*_ = 1		τ_*ex*_ = 5 ms	*w*_*max*_ = *g*_*max*_	
*p*_*elim*−*dep*_ = 0.0245		τ_*refract*_ = 5 ms		
*p*_*elim*−*pot*_ = 1.36*e*−4		τ_*inh*_ = 5 ms		
*f*_*rew*_ = 10 kHz		*V*_*reset*_ = *V*_*rest*_		
delay = 1 ms		*cm* = 20nF		
$\theta_{g}=\frac{1}{2}g_{\max}$				

We distinguish between two forms of rewiring: fast and slow. Fast rewiring consists of multiple attempts occurring every time step; at *f*_*rew*_ = 10 kHz 10 attempts are performed every time step. Conversely, slow rewiring would entail a synaptic rewiring attempted at an interval of several time steps; for example at *f*_*rew*_ = 100 Hz, 1 attempt would be performed every 10 time steps. All cores performing synaptic rewiring will be driven by a timer event. For each rewiring attempt, a single post-synaptic neuron is selected from the population using a random number generator (RNG) initialised with the shared seed. A synaptic element is then retrieved from SDRAM, the contents of which is decoded. The formation rule is followed if the synaptic element was empty, otherwise a synaptic removal is attempted. For the latter, the synaptic element contains sufficient information to identify the location of the appropriate synaptic row in SDRAM; the former requires the selection of one of the last received spikes to compute such an address. The address of the row is passed on to the DMA controller, which retrieves and stores it in DTCM where it can be operated upon. Once the required operation is performed, the row is written back to SDRAM and the process can commence once more.

The formation rule is considered when an empty synaptic element has been selected. It is at this point that Bamford et al. (2010) select the last neuron to have spiked as a partner pre-synaptic neuron. This approach is not applicable in our case because all the spikes within a time step are considered to be perfectly simultaneous, so the choice of a “last” spike is ill defined. If we indeed decide to define the “last” spike as the last received multicast packet we systematically skew the choice of partner toward neuron with higher identifiers within a partition. This effect occurs because SpiNNaker is a software simulator and iterates over neurons to resolve which ones are meant to fire in that specific time step. As a result, the neurons which are considered later within the time step are more likely to be selected by this naïve approach.

#### 2.2.3. Neural dynamics and synaptic plasticity

Neurons are simulated via a conductance-based leaky-integrate and fire (LIF) model, with the sub-threshold dynamics of an individual neuron evolving according to Equation 3:

(3) $$\begin{array}{lll} \tau_{m}\frac{\mathrm{dV}}{\mathrm{dt}} & = & V_{\mathrm{rest}}-V+g_{\mathrm{exc}}\left(E_{\mathrm{exc}}-V\right)+g_{\mathrm{inh}}\left(E_{\mathrm{inh}}-V\right) \end{array}$$

(4) $$\begin{array}{l} \frac{\text{dg}}{\text{dt}}=-\frac{g}{\tau_{s}}+\sum g_{\text{syn}}s_{i}\left(t-d_{i}\right) \end{array}$$

where *V* is membrane potential, τ_*m*_ the membrane leak time constant, *V*_*rest*_ the membrane resting potential, and *g* and *E* synaptic conductances and reversal potentials respectively. These synaptic conductances are considered to have been normalised by the membrane leak conductance, making them dimensionless quantities. When the membrane potential exceeds a threshold value *V*_*thr*_, a spike is emitted, and the membrane potential set to a reset potential *V*_*reset*_ for the duration of a refractory period τ_*refract*_. Synapses are either excitatory (*exc*) or inhibitory (*inh*), with both evolving in time (*t*) according to Equation 4. The second term on the right hand side represents the incoming spikes, with $s_{i}=\underset{k}{\sum }\delta \left(t-t_{k}^{i}\right)$ representing an incoming spike train from pre-synaptic neuron *i* at time *t* subject to delay *d*_*i*_. On receiving a spike a contribution of *g*_*syn*_ is added to the cumulative synaptic conductance *g*, before decaying with time constant τ_*s*_.

The STDP model uses an additive pair-based formulation to update *g*_*syn*_, with potentiation or depression depending on the relative timing of spike arrival at the pre and post-synaptic terminals Δ*t*_*STDP*_ = *t*_*post*_ − *t*_*pre*_. The classical formulation from Song et al. (2000) is used, as described in Equation 5:

(5) $$\begin{array}{l} g_{\text{syn}}=g_{\text{syn}}+f\left(\Delta t_{\mathrm{STDP}}\right)\\ \text{where }\text{f}\left(\Delta t_{\text{STDP}}\right)=\{\begin{array}{cc} A_{+}e^{\frac{\Delta t_{\text{STDP}}}{\tau_{+}}}\; & \mathrm{for}\;\Delta t_{\text{STDP}}>0\\ -A_{-}e^{\frac{\Delta t_{\text{STDP}}}{\tau_{-}}}\; & \mathrm{for}\;\Delta t_{\text{STDP}}<0\\ \; \end{array} \end{array}$$

where *A*_+_ and τ_+_ represent respectively the rate of learning and time constant for potentiation, and *A*_−_ and τ_−_ the same quantities for depression. Updates are performed to plastic synapses throughout a simulation whilst respecting the limits 0 ≤ *g*_*syn*_ ≤ *g*_*max*_.

#### 2.2.4. Experimental parameters

The experimental parameters match those used by Bamford et al. (2010), with exceptions detailed in Table 1. The first is that the simulation time step Δ*t* is 1 ms, c.f. 0.1 ms time step used in their model. This is a requirement if the desire is real-time simulation; a slow-down is imposed if the time step is less than 1 ms. A second difference consists in the presence of a refractory period τ_*refract*_ = 5 ms. By prohibiting the neuron from receiving synaptic input or spiking for this small time period we control its maximum firing rate, ensuring that the processing cores have ample time to process all of the events.

Connections all have a delay of one time step associated with them; *in silico*, post-synaptic neurons receive the spike almost instantaneously, but the effect of the spike is applied only after the specified delay. Moreover, PyNN distinguishes between the resting potential of a neuron and its reset potential; the former is the equilibrium level of the neuron, while the later is the level at which the membrane potential is set immediately following the generation of an action potential, afterwards decaying toward the resting potential as a function of the membrane leak time constant.

Input activity is generated by a layer of Poisson neurons. The firing protocol of these neurons depends on the simulation scenario, namely whether input correlations are present. When input correlations are not present in the input (Case 3), all *N*_*layer*_ pre-synaptic Poisson neurons produce a AER spike, on average, with a frequency of *f*_*mean*_ = 20 Hz. If input correlations are present in the input (Case 1 or Case 2 based on whether synaptic rewiring is enabled) the neurons exhibit differential firing rate. The firing rate is maximal at a randomly selected centre *s* on the grid of neurons and decreases with distance in accordance with Equation 6.

(6) $$\begin{array}{l} r\left(i\right)=f_{\text{base}}+f_{\text{peak}}e^{-\frac{\delta {\left(s,i\right)}^{2}}{2\sigma_{\text{stim}}^{2}}} \end{array}$$

where *r*(*i*) is the rate of Poisson neuron *i* = 1, …, *N*_*layer*_, δ(*s, i*) is the value of the Euclidean distance between the selected stimulus location *s* and a neuron *i* in the source layer, σ_*stim*_ is the desired spread of the stimulus, *f*_*base*_ is the base firing frequency or the level of noise applied to all neurons, onto which we add an exponentially decayed peak firing frequency *f*_*peak*_. A new location *s* is chosen at random every *t*_*stim*_ = 20 ms. Note that the equation, as reported, differs from the one in Bamford et al. (2010), which contains a typographical error (personal correspondence). We make use of the Gaussian-shaped input unless otherwise specified. In its current form, the overall firing rate of *f*_*mean*_ = 20 Hz is preserved.

We make use of three different types of initial connectivity. A rough topographic mapping is provided for validation (section 3.2) and is generated by following the formation rule (Equation 1) for each post-synaptic neuron using random pre-synaptic partners. This operation is performed until every neuron receives 16 afferents both from the source layer and from the target layer. Note that the same pair of neurons can (and most likely will) form multiple synapses (multapses) between them. As a consequence of this observation and the value of the peak lateral formation probability (*p*_*form*−*lat*_ = 1), the network will contain a number of synapses from a neuron to itself (autapses). Other experiments have been initialised with either one-to-one or fixed probability (*p* = 10%) connectivity.

In the paper by Song and Abbott (2001), B is defined as the ratio of the areas under the negative and positive portions of the STDP function:

(7) $$\begin{array}{l} B=\frac{A_{-}\tau_{-}}{A_{+}\tau_{+}} \end{array}$$

We follow Bamford et al. (2010) and use the same ratio regardless of projection (*B*_*ff*_ = *B*_*lat*_ = *B*) for computational tractability.

### 2.3. Analysis methodology

Topographic map quality is assessed using the measures defined by Bamford et al. (2010): the spread of the mean receptive field σ_*aff*_ and the deviation of the mean receptive field from its ideal location *AD*. The former relies on the search for the location around which afferent synapses have the lowest weighted variance ($\sigma_{\mathrm{aff}}^{2}$). This is a move away from the centre of mass measurement used by Elliott and Shadbolt (1999) for identifying the preferred location of a receptive field. As a result, the centre of the receptive field that is being examined is the location which minimises the weighted standard deviation, computed as follows:

(8) $$\begin{array}{l} \sigma_{\text{aff}}=\sqrt{\frac{\underset{i}{\sum }w_{i}|{\vec{p}}_{\mathrm{xi}}{|}^{2}}{\underset{i}{\sum }w_{i}}} \end{array}$$

where *i* loops over synapses, *x* is a candidate preferred location, $\left|{\vec{p}}_{\mathrm{xi}}\right|$ is the minimum distance from the candidate location to the afferent for synapse *i*, and *w*_*i*_ is the weight of the synapse. The candidate preferred location *x* has been implemented with an iterative search over each whole number location in each direction followed by a further iteration, this time in increments of 0.1 units. Thus, the preferred location $\underset{\vec{x}}{\arg\min}\sigma_{aff}$ of a receptive field is given by the function: $\arg\underset{\vec{x}}{\min}\sigma_{\mathrm{aff}}$.

Once the preferred location of each neuron is computed, taking the mean distance from the ideal location of each preferred location results in a mean Absolute Deviation (*AD*) for the projection. We report both mean *AD* and mean σ_*aff*_ computed with and without taking into account connections weights. σ_*aff*−*weight*_ and *AD*_*weight*_ are computed using synaptic weights *g*_*syn*_, while σ_*aff*−*conn*_ and *AD*_*conn*_ are designed to consider the effect of rewiring on the connectivity, thus synaptic weights are considered unitary.

These metrics are computed at different points throughout a simulation and have been suffixed accordingly: the suffix “init” refers to the initial state of the network, where all weights are initially maximized; “fin” refers to the final configuration of the network. Each of the results have an analog for comparison, suffixed with “shuf,” to be read as an abbreviation of “shuffle.” Shuffling in the context of weighted metrics (σ_*aff*−*weight*_ and *AD*_*weight*_) involves randomly reassigning synaptic weights, or shuffling them, among each neurons existing connections; in the context of the connectivity focused metrics (σ_*aff*−*conn*_ and *AD*_*conn*_) shuffling involves using the same rule that generated the initial synapses to re-generate the same number of synapses for each target neuron (Equation 1). The shuffled metrics are meant for comparison with the equivalent non-shuffled ones, i.e., mean σ_*aff*−*weight*_ is compared with mean σ_*aff*−*weight*−*shuf*_, mean *AD*_*conn*_ with mean *AD*_*conn*−*shuf*_ etc. These comparisons were performed using Wilcoxon Signed-Rank (WSR) tests on individual values of σ_*aff*_ and *AD* (for each neuron in the target layer) for a single simulation. We make use of a single initial rough topographic mapping used as the starting point for all simulations, except for those starting with one-to-one or fixed-probability connectivity.

A combination of multi-trial and single-trial metrics will be reported. One reason for reporting results over multiple trials is that inter-trial variability is a biological reality, and we strive to create spiking neural network simulations which can deliver sought after results by making use of the inherent stochasticity of such networks.

## 3. Results

### 3.1. Rewiring prunes depressed autapses

STDP has been a highly analyzed mechanism, with countless implementations *in silico* and empirical observations *in vivo* and *in vitro*. However, one type of behavioral use case has been overlooked, namely the behavior of STDP when a neuron projects to itself, also known as an autapse. Self-connections provide the neuron with no extra information, which is potentially a reason why they are avoided in brains from leeches to mammalians (Grueber and Sagasti, 2010). Self-avoidance occurs between neuronal processes originating from the same soma, thus allowing these neurites to spread out and not get entangled.

Spiking neural network simulators do not explicitly prohibit these kinds of self-connections, but their behavior might be undefined in some situations. For example, when defining delays on SpiNNaker they are interpreted as being entirely caused by the propagation of the signal down the dendrite to the soma, while the axon is modeled as propagating the spike instantly.

The existing STDP implementation on SpiNNaker was designed to include a propagation delay; a synapse will only see a post-synaptic spike caused by its cell after a delay (Stuart et al., 1997). Assuming that the dendritic delay is 1 ms (in both directions) and that a neuron forms a synapse with itself, a spike generated by the neuron at time *t*_*i*_ would instantly be made available to its autapse; this would be interpreted as a pre-synaptic action potential. However, the same synapse would be informed of the post-synaptic spike generated by its neuron at time *t*_*i*+1_. Thus, in Equation 5 Δ*t*_*STDP*_ = 0 and no change would occur at this point.

**Figure 2:**
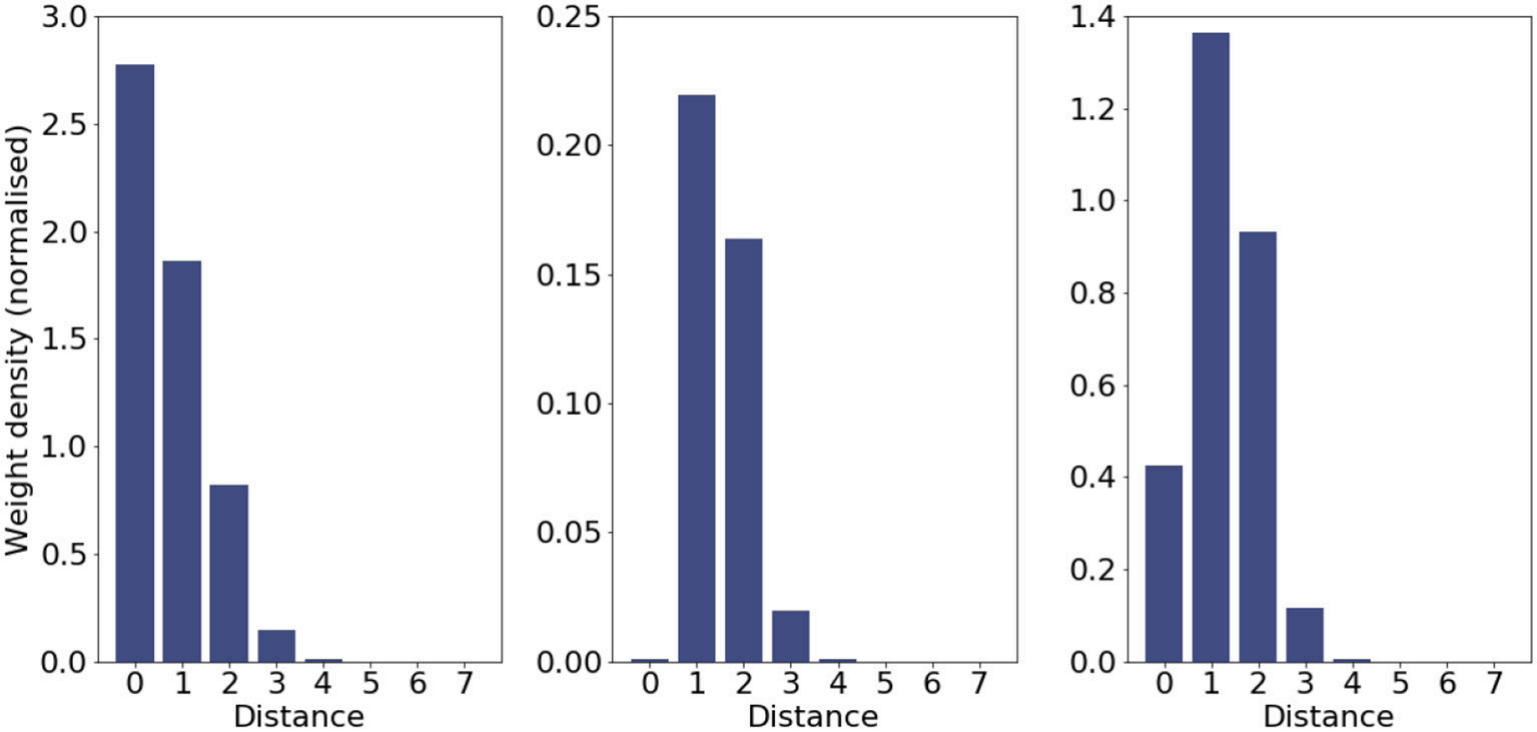
STDP behavior at an autapse and its influence on rewiring. The bar charts show the weight density of incoming lateral synapses (c.f. Figure 2 in Bamford et al., 2010) for the initial connectivity **(Left)**; final connectivity including weights **(Middle)**; and final connectivity with unit weights **(Right)**. The second plot shows that synapses at a distance of 0 (autapses) are maximally depressed, and the rewiring rule heavily prunes them.

However, the removal of the back propagation delay has the effect that the learning rule would maximally depress the autapse. Figure 2 shows that simply by removing this delay in propagation of information to the synapse we observe autapses being subjected to long term depression, prompting the synaptic rewiring rule eventually to prune them. These observations are purely for completeness, and the lack or presence of a delay back up the dendrite has no significant effect on the other presented results.

The behavior of the synaptic rewiring rule in this extreme case is to very aggressively prune the depressed autapses. Even in the presence of formation rule operating continuously, STDP guides the removal rule to free up the synaptic elements to be used for more useful connections; the preference against autapses is embedded into the connectivity of the network (last panel of Figure 2).

### 3.2. Validation

The three experiments performed by Bamford et al. (2010) are repeated here, where the network has the following 3 combinations of mechanisms acting upon it: STDP and synaptic rewiring, or exclusively STDP, both in the presence of input correlations (Case 1 and 2, respectively), and, finally, STDP in conjunction with synaptic rewiring, this time without input correlations (Case 3). The three aim to reveal the impact of input correlations and rewiring on the quality of the topographic maps (section 2.2.1 presents an overview and intuition of the model goals and operation). The initial connectivity is that of a fully formed, rough topographic mapping (section 2.2.4 describes its generation).

The single-trial results given in Table 2 (c.f. Table 2 in Bamford et al., 2010) are generated using the parameters described in section 2.2.4 and show that best results are achieved for the case in which both input correlations and rewiring are present in the network. Even without input correlations, the network with synaptic rewiring outperforms that relying solely on synaptic plasticity and input correlations, a result also shown by Bamford et al. (2010). Target-layer neurons have seen a reduction in the spatial spread of their receptive fields, first because STDP at their synapses has quickly adapted to the input statistics, but further on because the rewiring rule, guided by the STDP process, has modified the neuron's connectivity, thus further reducing the spread of the receptive field.

**Table 2:** Simulation results presented in a similar fashion to Bamford et al. (2010) for three cases, all of which incorporate synaptic plasticity.

**Case**	**1**	**2**	**3**
Synaptic rewiring	✓	✗	✓
Synaptic plasticity (STDP)	✓	✓	✓
Input correlations	✓	✓	✗
Target neuron mean spike rate	21.15 Hz	20.11 Hz	9.31 Hz
Final mean feedforward fan in / target neuron	15.91	N/A	11.87
Weight proportion of maximum	0.83	0.72	0.62
Mean σ_*aff*−*init*_	2.35	2.35	2.35
Mean σ_*aff*−*fin*−*conn*−*shuf*_	2.33	N/A	2.31
Mean σ_*aff*−*fin*−*conn*_	1.62	2.35	1.85
p(WSR σ_*aff*−*fin*−*conn*_ vs. σ_*aff*−*fin*−*conn*−*shuf*_)	2.80 × 10^−43^	N/A	3.65 × 10^−27^
Mean σ_*aff*−*fin*−*weight*−*shuf*_	1.61	2.32	1.78
Mean σ_*aff*−*fin*−*weight*_	1.49	1.92	1.57
p(WSR σ_*aff*−*fin*−*weight*_ vs. σ_*aff*−*fin*−*weight*−*shuf*_)	4.03 × 10^−33^	4.02 × 10^−43^	1.44 × 10^−21^
Mean *AD*_*init*_	0.81	0.81	0.81
Mean *AD*_*fin*−*conn*−*shuf*_	0.82	N/A	1.09
Mean *AD*_*fin*−*conn*_	0.77	0.81	0.91
p(WSR *AD*_*fin*−*conn*_ vs. *AD*_*fin*−*conn*−*shuf*_)	0.39	N/A	0.002
Mean *AD*_*fin*−*weight*−*shuf*_	0.79	0.92	1.04
Mean *AD*_*fin*−*weight*_	0.85	0.79	1.07
p(WSR *AD*_*fin*−*weight*_ vs. *AD*_*fin*−*weight*−*shuf*_)	0.0002	0.0001	0.58

*Case 1 consists of a network in which both synaptic rewiring and input correlations are present. Case 2 does not integrate synaptic rewiring, but still has input correlations. Case 3 relies on synaptic rewiring and STDP to generate sensible topographic maps in the absence of input correlations*.

**Figure 3:**
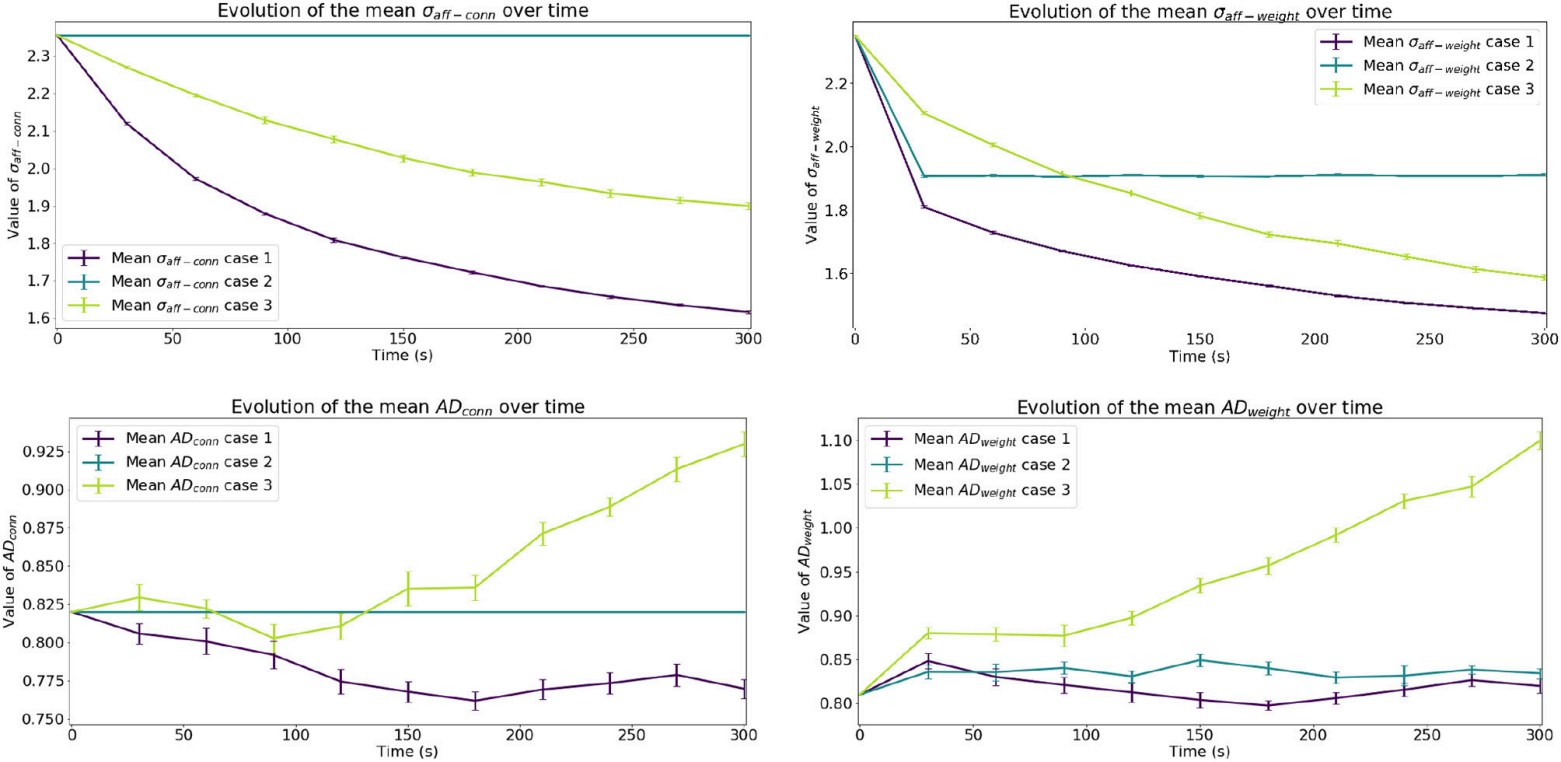
Evolution of results of interest. The top row shows the evolution of the mean spread of receptive fields over time, considering both unitary weights (σ_*aff*−*conn*_) and actual weights (σ_*aff*−*weight*_) at that point in time. The bottom row shows the evolution of the mean Absolute Deviation of the receptive fields considering connectivity (*AD*_*conn*_) and weighted connectivity (*AD*_*weight*_). Error bars represent the standard error of the mean.

Figure 3 shows the evolution in time of this effect in multiple simulations. When rewiring is present, the spread of the receptive fields σ_*aff*_ tends to decrease slowly to a smaller value, but changes driven solely by STDP settle more quickly. Even though we have increased the rate at which rewiring happens artificially (*f*_*rew*_ = 10 kHz), the time constants involved are smaller for STDP. This difference in time constants is revealed in the rate of change of σ_*aff*−*weight*_ compared to σ_*aff*−*conn*_; the weighted refinement process precedes the reduction in spread of receptive field at the level of connectivity. The increase in mean *AD* in Case 3 is symptomatic of an instability in the network and is addressed in section 3.4.

**Figure 4:**
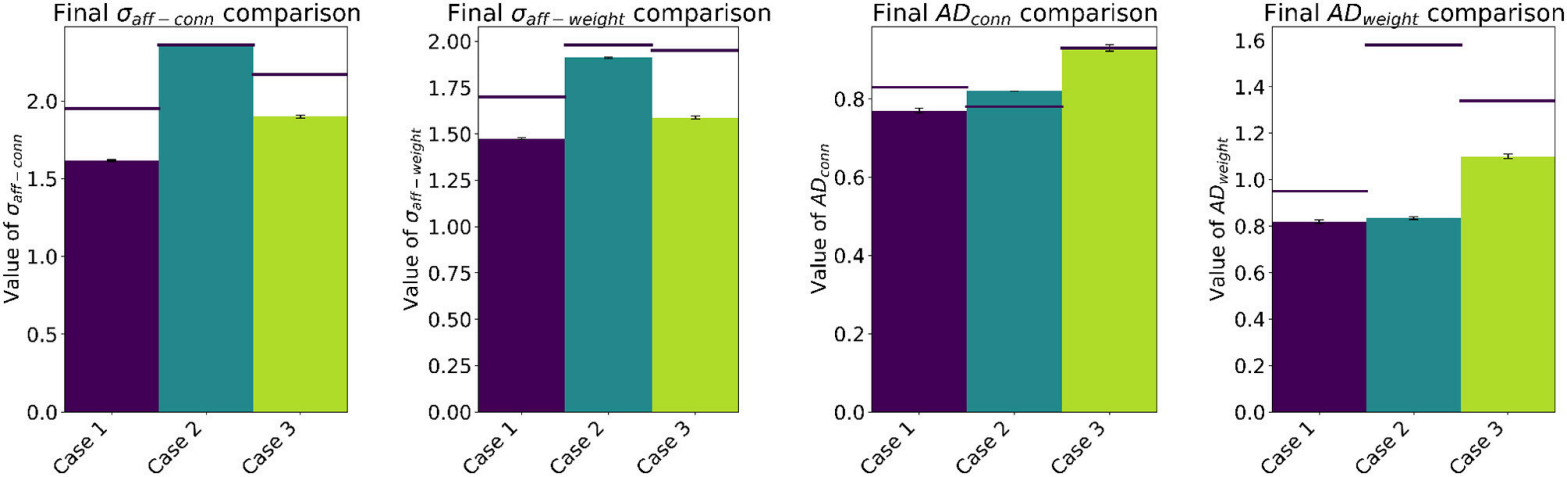
Final values of σ_*aff*−*conn*_, σ_*aff*−*weight*_, *AD*_*conn*_ and *AD*_*weight*_, including standard error of the mean computed from values obtained over 10 experiments. Horizontal lines show single-trial results from Bamford et al. (2010).

**Figure 5:**
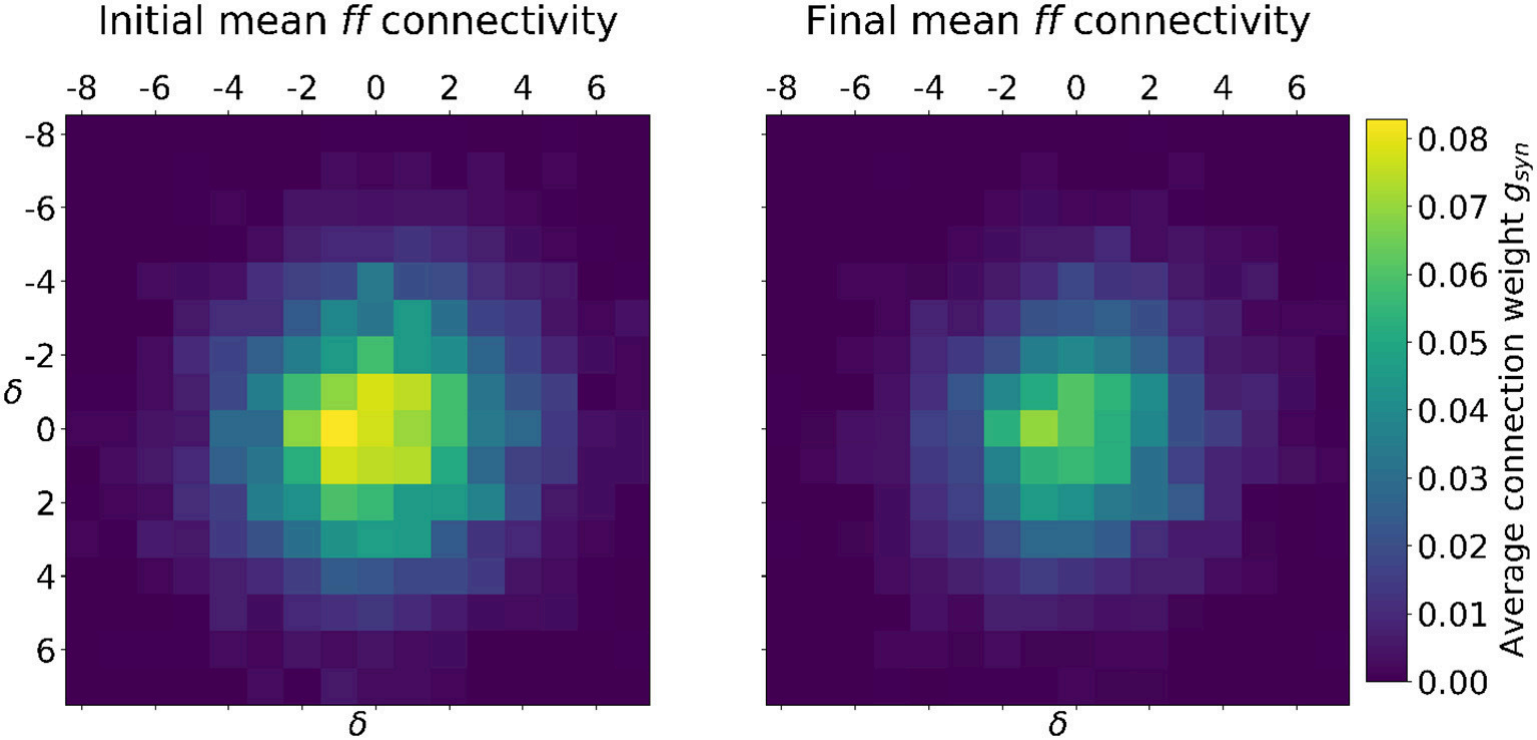
Example Case 2 initial **(Left)** and final **(Right)** distributions of mean weight at discrete relative distances from individual neurons. STDP sharpens the receptive field by depressing synapses far from stimulus centers. For reference, the initial connectivity **(Left)** started with a σ_*aff*−*init*_ = 2.35 and an *AD*_*init*_ = 0.81, values which were refined by the end of the simulation **(Right)** to σ_*aff*−*weight*_ = 1.92 and an *AD*_*weight*_ = 0.79. Table 2 contains additional reported parameters.

A comparison between our multi-trial results and the single-trial values reported in Bamford et al. (2010) is presented in Figure 4. The results are generally in good agreement, apart from the Case 2 value of *AD*_*weight*_ which shows a factor of ≈2 difference between the two studies. Although there are slight differences between the two underlying models, such as the use of a refractory period in this work, it is not thought they are the cause of this variation. Inspection of the initial and final values of *AD*_*weight*_ in Bamford et al. (2010) shows that for Case 2 the final configuration is significantly worse than the initial conditions, despite the constant value of *AD*_*conn*_ due to the fixed connectivity. Whereas in this work, Case 2 sees STDP refine the initial weight matrix, and sharpen the receptive field about the stimulus—as demonstrated in Figure 5 detailing the Case 2 initial and final weight distributions. This behavior is as expected, where STDP will depress synapses distant from the stimulus center due to reduced correlation between the stimulus and output spikes. It is believed the implementation in Bamford et al. (2010) may have suffered from an increased bias toward synaptic depression, which in turn led to deviation of the receptive field center due to the lack of strongly weighted synapses to anchor it about the idealized initial condition. The improved performance of STDP in this work is therefore thought to contribute to the reduced Case 1 and 3 values of σ_*aff*−*conn*_ and σ_*aff*−*weight*_ shown in Figure 4, where both rewiring and STDP are active. Note that the improvement for Case 1 is greater due to the presence of input correlations.

### 3.3. Development of topographic maps

Modeling developmental formation of topographic maps using the presented mechanisms could yield some interesting results with regard to total speed of simulation and could be used to validate whether the network can still generate good quality topographic maps. Regarding the former, simulations with far more neurons and synapses could benefit from not requiring a fixed connectivity be loaded onto SpiNNaker—the process of interacting with SpiNNaker for loading or unloading data is currently the main bottleneck. The latter is meant as a validation for the model, but also to gain additional insights into its operation, specifically whether maps formed through a process of simulated development react differently to maps which are considered in their “adult,” fully-formed state in need of refinement.

**Figure 6:**
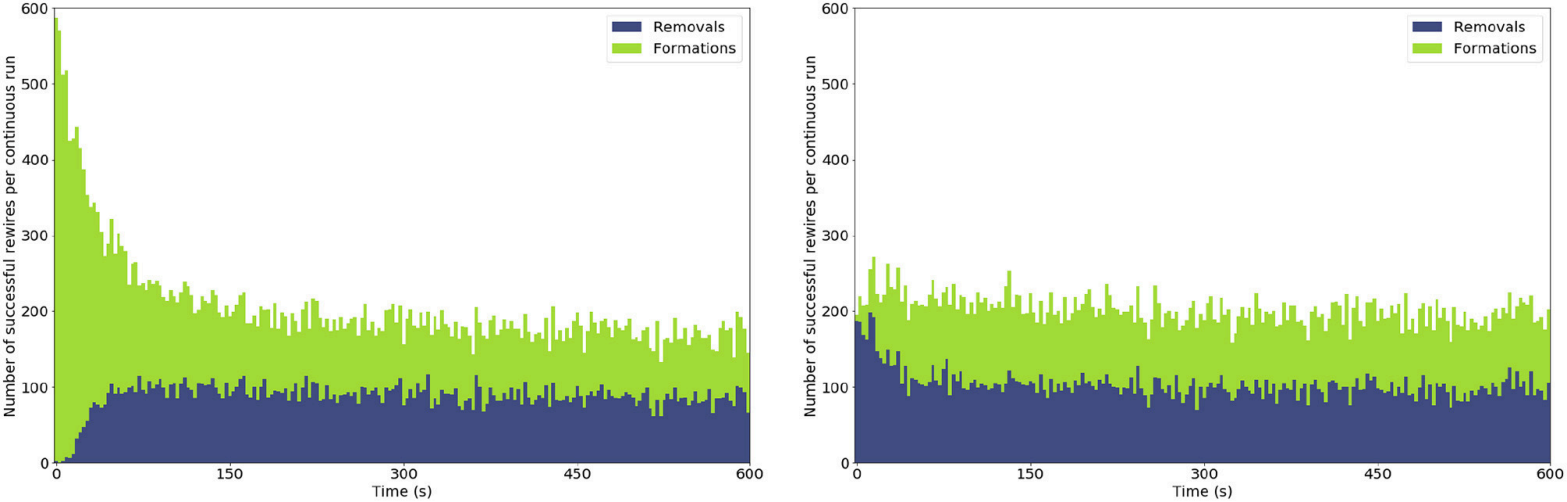
Stacked bar chart of formations and removals over time within one simulation. **(Left)** evolution of the network starting from no connections; **(Right)** evolution of the network starting from a sensible initial connectivity. The number of formations or removals is aggregated into 3 s chunks.

Results from this simulation are presented in tabular form (Table 3), with the addition of longitudinal snapshots into the behavior of the network and mean receptive field spread and drift, as well as a comparison between different initial connectivity types (topographic, random percentage-based and minimal). In the initial stages of the simulation the σ_*aff*_ and *AD* are almost zero due to the lack of connections, but they steadily increase with the massive addition of new synapses. Figure 6 shows a side-by-side comparison of the number of rewires between of development or adult refinement. The developmental model initially sees a large number of synapses being formed until an equilibrium is reached at around 10% connectivity. A 10% connectivity is also achieved when starting the network from an adult configuration. This does not mean that every set of parameters will yield the same result. In this case, and all the others in this paper, we locked the maximum fan in for target layer neurons to 32, or 12% connectivity. As a result, the network is bound to have at most that connectivity and at least half that, or 6%, if formation and removal occur with equal probability.

**Table 3:** Results for modeling topographic map formation from development (minimal initial connectivity).

**Case**	**1**	**3**
Synaptic rewiring	✓	✓
Synaptic plasticity (STDP)	✓	✓
Input correlations	✓	✗
Target neuron mean spike rate	18.49 Hz	9.80 Hz
Final mean fan in / target neuron	17.69	11.92
Weight proportion of maximum	0.83	0.63
Mean σ_*aff*−*init*_	0	0
Mean σ_*aff*−*fin*−*conn*−*shuf*_	2.39	2.30
Mean σ_*aff*−*fin*−*conn*_	1.56	1.67
p(WSR σ_*aff*−*fin*−*conn*_ vs. σ_*aff*−*fin*−*conn*−*shuf*_)	1.14 × 10^−43^	2.27 × 10^−35^
Mean σ_*aff*−*fin*−*weight*−*shuf*_	1.56	1.59
Mean σ_*aff*−*fin*−*weight*_	1.44	1.26
p(WSR σ_*aff*−*fin*−*weight*_ vs. σ_*aff*−*fin*−*weight*−*shuf*_)	1.25 × 10^−36^	6.21 × 10^−31^
Mean *AD*_*init*_	0	0
Mean *AD*_*fin*−*conn*−*shuf*_	0.79	0.99
Mean *AD*_*fin*−*conn*_	0.64	0.85
p(WSR *AD*_*fin*−*conn*_ vs. *AD*_*fin*−*conn*−*shuf*_)	1.3 × 10^−4^	0.01
Mean *AD*_*fin*−*weight*−*shuf*_	0.66	1.02
Mean *AD*_*fin*−*weight*_	0.65	0.96
p(WSR *AD*_*fin*−*weight*_ vs. *AD*_*fin*−*weight*−*shuf*_)	0.84	0.51

**Figure 7:**
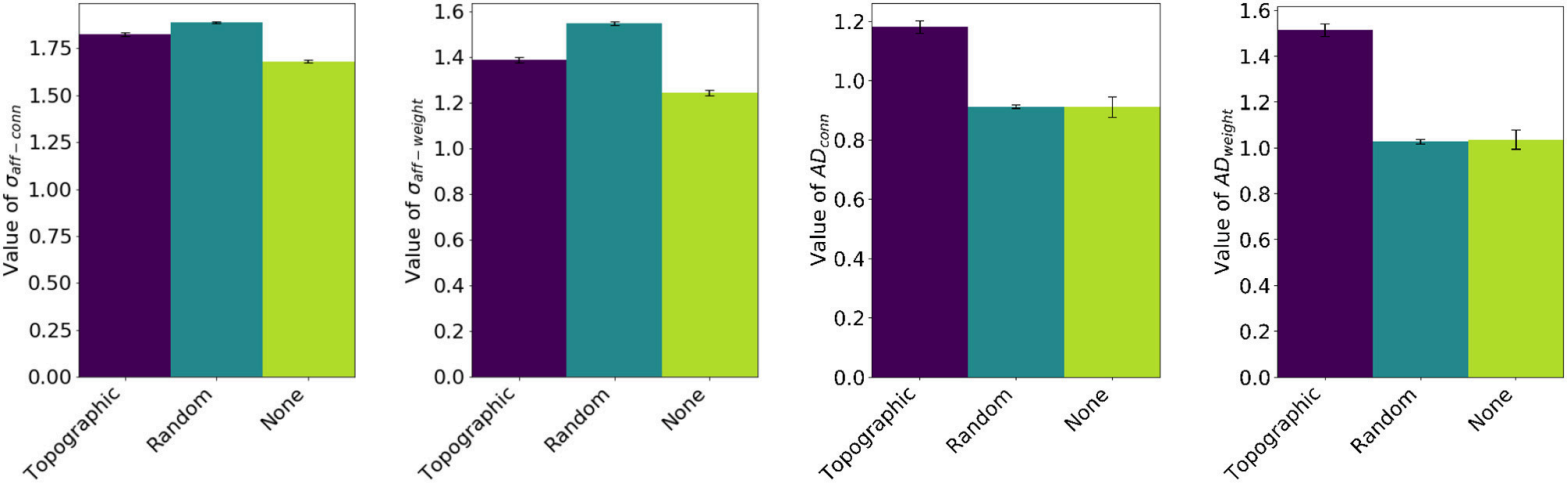
Comparison of final values for σ_*aff*_ and *AD* in the case where input correlations are absent (Case 3). Three types of networks have each been run 10 times (to generate the standard error of the mean), each starting with a different initial connectivity: an initial rough topographic mapping as in the previous experiments, a random 10% connectivity (5% feedforward, 5% lateral) and almost no connectivity (one-to-one connectivity used due to software limitations).

Table 3 shows the final, single-trial, results for a network identical to previous experiments, but with a run time of 600 s. This ensures the networks have a chance to converge on a value of σ_*aff*_ and AD. A comparison between Tables 2, 3 shows similar results for case 1, but significantly better results for case 3. These differences are summarized in Figure 7. σ_*aff*_ and *AD* were computed at the end of three simulations differing only in the initial connectivity: an initial rough topographic mapping as in the previous experiments, a random 10% initial connectivity balanced between feedforward and lateral, and almost no initial connectivity (in practice, one-to-one connectivity was used due to software limitations). We do not simulate the case without synaptic rewiring, as the results would be severely impacted by the lack of rough initial topographic mapping. The final mean value of *AD* is improved in both experiments involving initial non-topographic connectivity and σ_*aff*_ is improved when the network starts with no initial connectivity.

### 3.4. Stable mappings arise from lateral inhibition

Following the previous results, coupled with the tendencies exhibited by the results generated for Case 3 in Figure 3, further simulations have been run to confirm whether input correlations are necessary to generate stable topographic maps. Our results are inconclusive as although the weighted Absolute Deviation of the mean receptive field increased to 2.25, or 1.64 if only considering connectivity, the final mean number of feedforward synapses reduces to around 5, making these statistics unusable.

**Figure 8:**
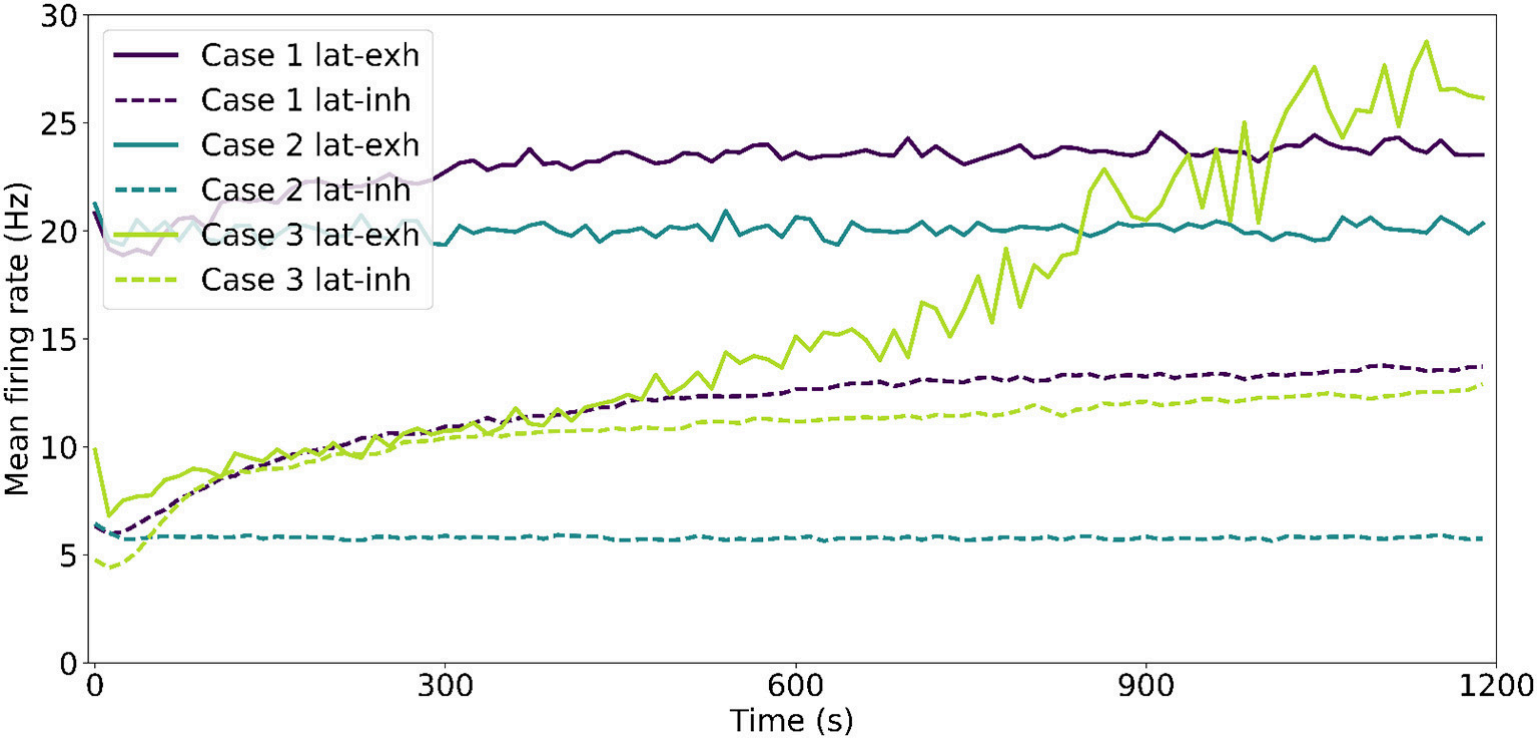
Target layer firing rate evolution throughout the simulation. The instantaneous firing rate has been computed in 1.2 s chunks for simulations where lateral connections are excitatory (lat-exh) and for simulations where lateral connections are inhibitory (lat-inh).

We hypothesize that since the feedforward is reduced so heavily, both in terms of connectivity (on average 5 incoming connections for each target-layer neuron) and in terms of synaptic weights (0.3 of maximum possible for the present connectivity) that the lateral connections within the target layer drive the comparatively high activity in the target layer (Figure 8). This, in turn, causes the pre-synaptic partner selection mechanism to focus its attention mostly on the target layer. We have achieved a reduction in the target layer firing rate by introducing inhibitory lateral connections. This is sufficient to generate a stable topographic mapping which matches quite closely the results of the original network when both input correlations and synaptic rewiring are present: σ_*aff*−*conn*_ = 1.74, σ_*aff*−*weight*_ = 1.38, *AD*_*conn*_ = 0.85, *AD*_*weight*_ = 0.98; all results are significant. The combined choice of sampling mechanism and lateral inhibition has a homeostatic effect upon the network.

Conversely, in the cases where input correlations are present, we see stable topographic mapping, regardless of the presence of synaptic rewiring, as well as significantly more feedforward synapses. Finally, no applicable network was negatively impacted by initializing the connectivity either randomly or with minimal connections.

To sum up, the model can generate transiently better topographic maps in the absence of correlated input when starting with a negligible number of initial connections or with completely random connectivity. These results can also be stabilized with the inclusion of lateral inhibitory connections, which can prevent self-sustained waves of activity within the target layer. Experiments which included correlated inputs do not require inhibitory lateral feedback either for reducing the spread of the receptive field or for maintaining a stable mapping. Finally, the model has proven it is sufficiently generic to accommodate changes in initial connectivity, as well as type of lateral connectivity i.e., the change from excitatory to inhibitory synapses.

### 3.5. Sensitivity analysis

Two key advantages arise from running spiking neural network simulations on SpiNNaker: some large or complex simulations can be simulated in real-time and there is very high potential for horizontal scalability of simulations through massive parallelism; the latter is due, in part, to the physical size of the machine comprising 500,000 cores (for details see section 1.3). This implementation demonstrates feasibility and correctness of synaptic rewiring on a small network with relatively short run times. This successful implementation forms a foundation for future work investigating applications with more biologically realistic learning rates and at larger scale. Sensitivity analysis represents another application in which both features can be taken advantage of to extract insights into models. Simulations are independent, thus can be run in parallel threads of a host central processing unit without the risk of deadlock or the need for file sharing. The half a million core SpiNNaker machine is also capable of being partitioned into smaller independent systems dynamically at run time.

The model for topographic map refinement proposed by Bamford et al. (2010) contains a non-trivial number of parameters; they are a combination of sensible defaults, experimental results from literature and values chosen empirically. A more structured approach into understanding the influence of parameters and various network configurations on the quality of topographic maps is presented herein. Two questions are tackled: what is the model resilient to in parameter space? and conversely: what is the model sensitive to in parameter space?

Our exploration begins from the experimental parameters presented in section 2.2.4—and will revolve around two areas of network: STDP and input characteristics.

The premise of employing synaptic rewiring for modeling topographic map formation is that synapses on the edge of the receptive fields of neurons will tend to be depressed because of the combined effect of input correlations and lateral feedback. At that point, synaptic rewiring is free to re-use that synaptic terminal to form a new connection that can prove more useful. We explore the effect of varying the level of depression in the network through two parameters: B and τ_−_.

**Figure 9:**
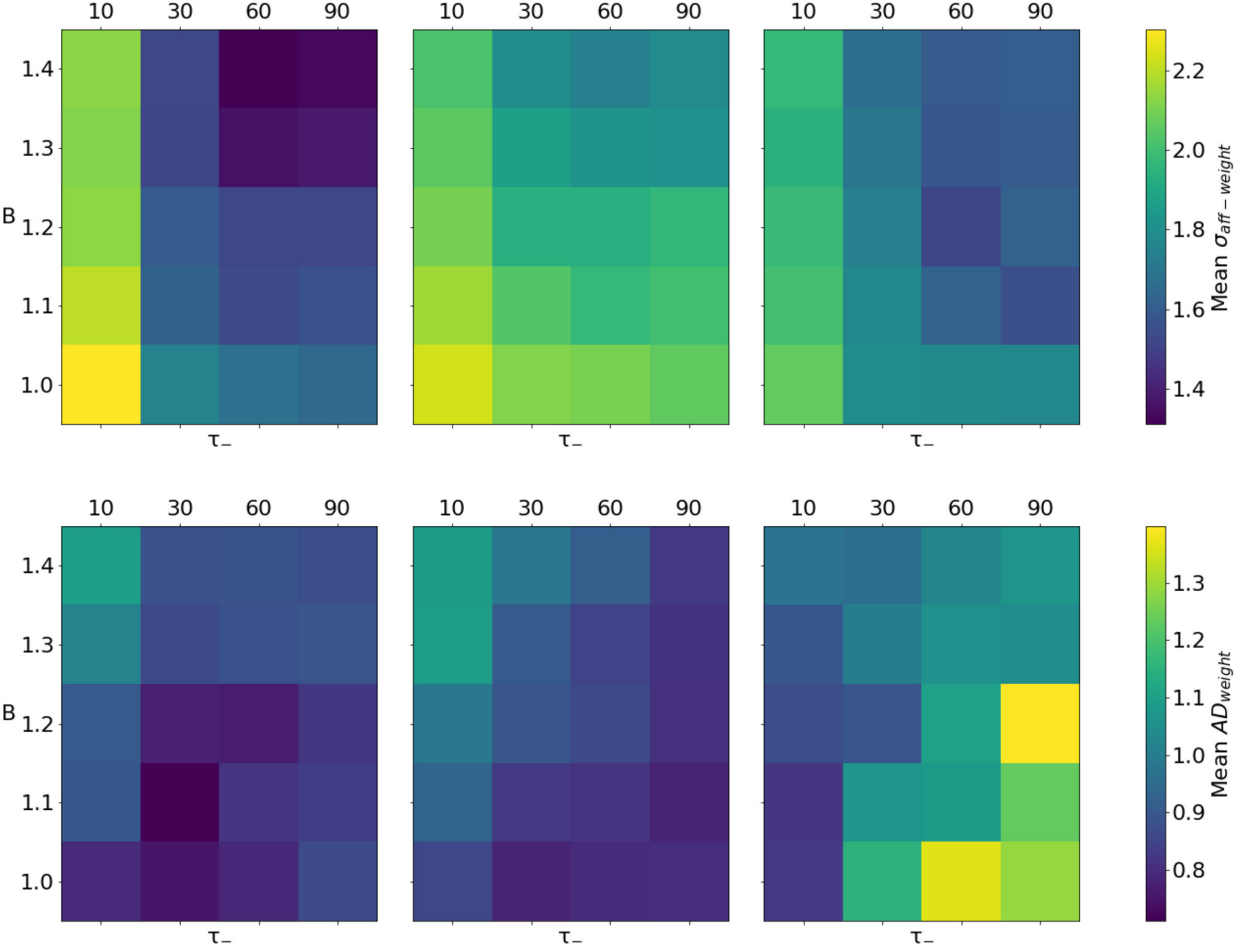
Quality of topographic maps when varying the ratio between inhibition and excitation (B, see Equation 7) and the depression time constant (τ_−_). Lower values are better. **Top row**: mean spread of the afferent receptive field σ_*aff*−*weight*_. **Bottom row**: mean weighted Absolute Deviation of the centres of the receptive fields *AD*_*weight*_. Columns represent, from left to right, the 3 considered cases: STDP and synaptic rewiring, or exclusively STDP, both in the presence of input correlations (Case 1 and 2, respectively), and, finally, STDP in conjunction with synaptic rewiring, this time without input correlations (Case 3).

Figure 9 shows the effect of varying these parameters on the 3 cases of interest. The values have been computed from a single run with the combination of parameters indicated by the row and column. Here we focus on the weighted metrics (σ_*aff*−*weight*_ and *AD*_*weight*_) as they are a good indicator of network behavior. Best results for σ_*aff*−*weight*_ are achieved when a long depression time constant is used in conjunction with a larger depression weight change. This trend is present in the other cases, but with less pronounced effects. In terms of *AD*_*weight*_, the trends suggest that an equal amount of depression and potentiation is preferable, except when the time constant for depression τ_−_ is longer and inputs are completely uncorrelated.

**Figure 10:**
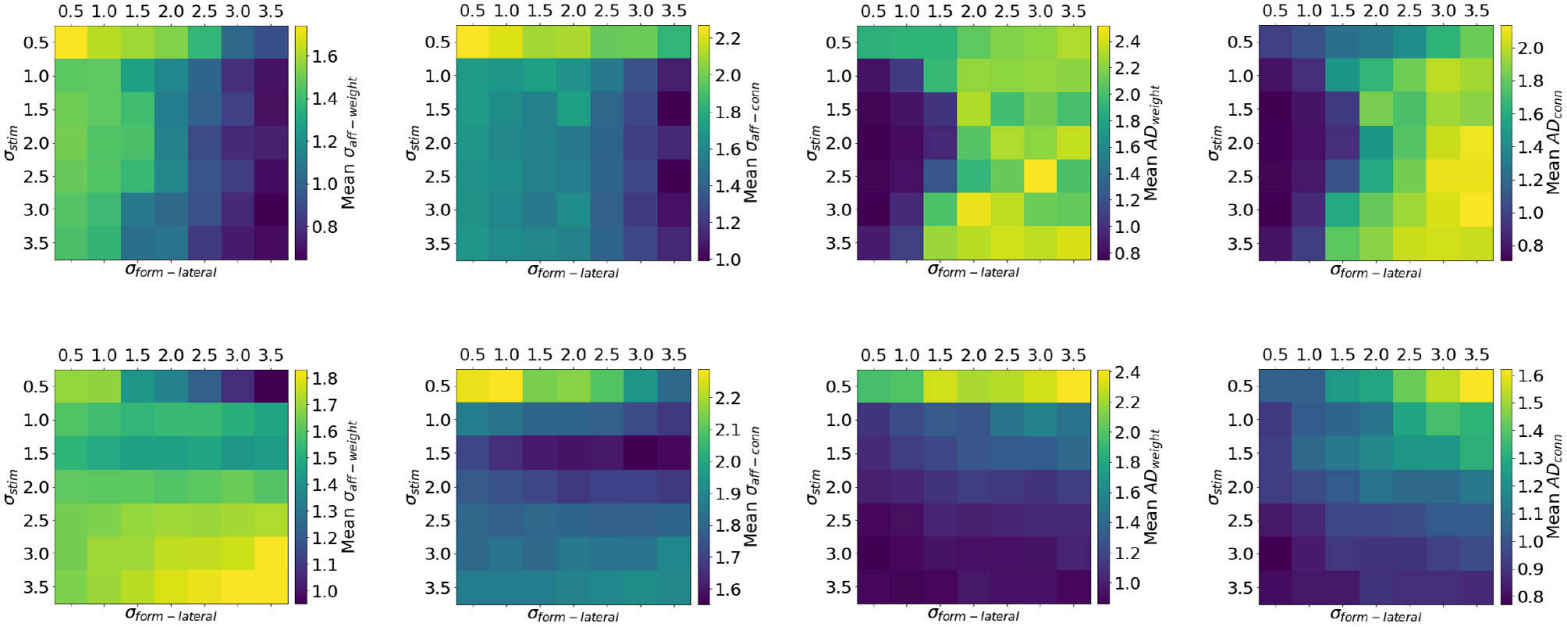
Quality of topographic maps when varying the spread of the stimulus σ_*stim*_ and σ_*form*−*lat*_ (experimental setup of Case 1). The top row shows results from simulations wherein lateral connections are excitatory, while in the bottom row lateral connections were inhibitory. When lateral connections are inhibitory the network is less sensitive to the choice of σ_*form*−*lat*_ compared to having excitatory connections. Moreover, lateral inhibition stabilizes the mapping.

The top row of Figure 10 shows the effect of co-varying σ_*stim*_ and σ_*form*−*lat*_ with otherwise default parameters and in the presence of input correlations. The default choice of parameters (σ_*stim*_ = 2 and σ_*form*−*lat*_ = 1) lands in a space where *AD* is stable and the reduction in variance of the mean receptive field is significant. However, for values of σ_*form*−*lat*_ greater than 2, the topographic mapping is unstable because of the reasons provided in section 3.3. Indeed, for these pairs of parameters the firing rate of the target layer is self-sustaining. The introduction of lateral inhibition once again stabilizes the mapping (bottom row of Figure 10). The network is now less sensitive to the choice of σ_*form*−*lat*_ both in terms of σ_*aff*_ and *AD*. The exception in this case is when considering an input with very low spatial variance (σ_*stim*_ = 0.5). The network is not capable of significantly reducing the variance of the connectivity-only receptive field, either with excitatory or inhibitory lateral connections. The *AD* is also negatively impacted in this case. One explanation for this effect is that, due to the size of the input variance σ_*stim*_ = 1, the input stimulus is dominated by a single neuron firing at around 2.3 kHz (in order to maintain the mean firing rate *f*_*mean*_ = 20 Hz); this can only drive the STDP mechanism to perform long term-depression on all synapses which see this activity (the network favors depression when *B* > 1).

Sensitivity analysis can be a powerful tool for gaining deeper understanding of networks where complex interactions are at play. In the context of SpiNNaker, this type of analysis can prove extremely useful, especially when running very large simulations which can be accelerated by the hardware. In our case, it has revealed that biasing the STDP mechanism toward depression, both in terms of strength and time constant, can improve the quality of topographic maps. Values of 1.3 ≤ *B* ≤ 1.4, combined with time constants for depression of 60ms ≤ τ_−_ ≤ 90ms yield best results for σ_*aff*−*weight*_ and *AD*_*weight*_.

Finally, lateral inhibition is preferred over excitation as it stabilizes the topographic projection and allows the network to be less sensitive to input size or the spread of the lateral receptive fields. While the presence of very long range lateral excitatory connections (σ_*form*−*lateral*_ ≥ 3) yields lowest values of σ_*aff*_ these mappings are unstable. However, longer-range lateral inhibition (σ_*form*−*lateral*_ ≥ 2.0) results in stable mapping in all but one input configuration: where the input is punctiform (σ_*stim*_ = 0.5) and firing at more than 2 kHz. Best results here are achieved when the input 1 ≤ σ_*stim*_ ≤ 2.

## 4. Discussion

We described the design and implementation of a model of synaptic rewiring simulated on the SpiNNaker neuromorphic platform using PyNN as the network description language. Moreover, we showed that the model can be simulated in real-time, allowing for longer runs modeling development or multiple runs to explore the sensitivity of the network. The provided validation and the open-source nature of the simulator and of the network description means that our effort can be easily interrogated or extended.

We showed that continuously operating STDP and synaptic rewiring can refine a topographic map regardless of the initial mapping between neurons. Running simulations for longer revealed a possible source of instability in the network, namely the excitatory lateral connections. Under certain conditions, self-sustaining waves of activity occurred in the target layer, resulting in STDP favoring lateral connections over feedforward ones. We replaced the excitatory connections with inhibitory ones and immediately observed the homeostatic and stabilizing effect they had in conjunction with the sampling mechanism. Sensitivity analysis revealed operational ranges of STDP, rewiring and input parameters.

In the following sections we discuss computational characteristics of SpiNNaker in relation to the model of synaptic rewiring at hand. Furthermore, we make an argument on the efficiency of the implemented structural plasticity framework. Finally, we present a suite of future extensions and uses of the work presented herein.

### 4.1. Performance

This was the first attempt at structural plasticity on SpiNNaker. Moreover, this mechanism was never considered in the design process of the system, neither hardware, nor software. However, it has turned out that the complete neuromorphic package has been sufficiently flexible to accommodate synaptic rewiring in parallel with STDP in real time.

Framework scalability can be interpreted from the point of view of individual processing cores (vertical scaling) or from the point of view of all available processing cores (horizontal scaling). Horizontal scaling is currently limited by the amount of additional metadata that needs writing in core-local memory and the entries present in the routing tables (router current assumes all-to-all. The exponential decay lookup tables discussed at the end of section 2.2.2 are dependent on (1) the size of the layers and (2) the magnitude of standard deviations used for formation σ_*form*−*ff*_ and σ_*form*−*lat*_. The amount of metadata which needs to be added on chip could be reduced by pruning the information pertaining to impossible connections. Rinke et al. (2016) suggest an efficient algorithm for neuron selection during synaptic rewiring based on *n*-body problems, where pairs of bodies have to be considered for force calculations. Their approximation technique relies on observations that particles sufficiently far away from a target particle need not be considered individually. They apply this algorithm to the model proposed by Butz and van Ooyen (2013), but it could be applied here for pruning routes which will never materialise into synaptic connections.

Vertical scalability depends on a few characteristics of the networks, but also on some of the model parameters. SpiNNaker processing cores operate at approximately 200 MHz and have small amounts of attached memory, capable, in theory, of simulating at most 1,000 neurons. Additional processes executing on a core, such as STDP, have the effect of reducing the number of simulated neurons significantly, especially when operating under the real time execution constraint. The requirement that rewiring be performed at a fixed rate requires additional retrieval of information from SDRAM than is otherwise necessary in normal operation. In the current implementation (detailed in section 2.2.2) a DMA read is required to retrieve the synaptic row of a pre-synaptic neuron to DTCM. This is an extra read operation in excess of the read operations performed whenever a spike is received. A spike-driven rewiring process could make use of the existing infrastructure and only operate on synaptic rows which are in core-local memory.

### 4.2. On efficiency

The existing sPyNNaker framework for the implementation of neural networks on SpiNNaker supports user extensions in the form of new neuron models and new STDP plasticity weight update rules. The work presented in this paper extends this framework to support implementation of new structural plasticity rules. The use of frameworks when using neuromorphic systems such as SpiNNaker allows for efficient integration of new rules without requiring users first understand the existing code or even the underlying hardware and execution model. This is because the integration points are chosen specifically to execute in the most efficient way on the platform, and present interfaces to be filled in by the users which reflect this point of integration. For example, code which modifies synaptic connectivity information on SpiNNaker (such as changes in weight as performed by STDP) is best executed whilst the synaptic data is in the core local memory (DTCM); in normal operation of the software this occurs whilst processing an incoming spike. Thus STDP update rules are written to operate whilst an incoming spike is being processed, with appropriate information being cached until this is the case.

In terms of structural plasticity, additional information is made available to allow updates to be performed on a time-step basis outside of the processing of incoming spikes. This information has been provided in a compact form that works well within the current software framework, and so can now be used by any other structural plasticity formation or deletion rule without having to be implemented a second time. This also ensures correctness as the synaptic information is copied from SDRAM during processing, and written back once processing is complete, the software must ensure that a second copy of SDRAM is not made until the first has been processed and written back. This is particularly important where STDP and structural plasticity may both wish to modify the same part of the synaptic matrix at the same time; the event-based nature of the execution of SpiNNaker code, and the use of the DMA engine to transfer data between DTCM and SDRAM in parallel to code execution on the CPU make it easy to get into an inconsistent state if the implementation is not carefully done. The use of the framework developed here helps avoid these mistakes in future structural plasticity implementations.

### 4.3. Future work

An immediate imperative is addressing inherent scalability issues, as discussed in the previous section. Modeling larger neuronal layers could prove fruitful to investigate the behavior of the model with more realistic inputs, such as those originating from an event-based dynamic vision system and representing both natural and artificial scenes. Such models would necessarily also be simulated on longer time scales than presented herein so as to allow sufficient time for neurons to adapt to the statistics of the input. A move toward more natural input could consist of handwritten digits represented as spike trains (MNIST, LeCun et al., 1998; Liu et al., 2016). Classification could be performed using a network comprised of separate maps sensitized to different digits connected to a winner-takes-all circuit.

The framework as implemented is a platform-dependent extension of the PyNN specification. More community input could drive the modification of the PyNN application programming interface (API) to natively support multiple models of structural plasticity.

Following evidence of topographic projections being stabilized in the presence of lateral inhibition, an extension to the model including both inhibitory and excitatory lateral connectivity could similarly be implemented and analyzed. Moreover, all the aforementioned network architectures could be extended to include distance-based synaptic delays; spatio-temporal patterns could then be embedded into the network connectivity. An additional extension could see multiple formations or removals occurring each rewiring attempt, which could decrease the refinement time of the topographic mapping. This approach could be driven by a different type of event: the reception of an AER spike, rather than a timer interrupt because the synaptic information will be available in core-local memory.

Finally, future work could also focus on choosing a different mechanism for selecting pairs of neurons as partners for formation. Preliminary results show little qualitative differences between a random selection of pre-synaptic partner for formation and the selection based on later spike times. However experiments have not been run for simulations longer than 300 s or when varying parameters.

## Author contributions

AR provided input into the design and implementation of the structural plasticity framework and model. OR presented the current learning developments on the SpiNNaker platform and created a more efficient implementation of a variable-rate Poisson spike source. SF contribution lies with understanding the model, how it is affected by the hardware and debugging the behavior of the network simulations. PB worked on all of the above.

### Conflict of interest statement

The authors declare that the research was conducted in the absence of any commercial or financial relationships that could be construed as a potential conflict of interest.

## Footnotes

^1^The data resulting from running simulations, scripts describing the networks in their entirety, plotting and analysis of the data are available at the following repository: http://dx.doi.org/10.17632/xfp84r5hb7.1
